# Complementary killing activities of *Pbunavirus LS1* and *Bruynoghevirus LUZ24* phages on planktonic and sessile *Pseudomonas aeruginosa* PAO1 derivatives

**DOI:** 10.1128/aac.00579-25

**Published:** 2025-07-23

**Authors:** Maud Billaud, Clarisse Plantady, Benoît Lerouge, Emma Ollivier, Julien Lossouarn, Elisabeth Moncaut, Julien Deschamps, Romain Briandet, Aurore Cleret, Cindy Fevre, Gaëlle Demarre, Marie-Agnès Petit

**Affiliations:** 1 Université Paris-Saclay, AgroParisTech, INRAE, MICALIS 27048, Jouy en Josas, France; 2 Phaxiam Therapeutics 455297 , Lyon, France; University of Fribourg, Fribourg, Switzerland

**Keywords:** bacteriophage, *psl*, *fklB*, *rocS1*, *wzy*, biofilm

## Abstract

Four phages active against a representative panel of *Pseudomonas aeruginosa* strains were chosen with the goal of using them for phage therapy. Two were belonging to the *Pbunavirus LS1* species, and two to the *Bruynoghevirus LUZ24* species. The receptor of the *P. LS1* phage Ab27 had already been characterized as the O-antigen chain of the lipopolysaccharides, whereas no information was available at the onset of this work on the receptor used by the *B. LUZ24* phages. We show that this receptor is the surface polysaccharide Psl, an important component of the biofilm matrix. Remarkably, the *B. LUZ24* phages were more active against PAO1 in minimal medium compared to rich medium. This was correlated with larger amounts of Psl bound at the bacterial surface during exponential growth in minimal medium, compared to the rich medium. Phages prevented biofilm growth when applied early after biofilm formation on a medical endotracheal tube, as well as in 96-well plates, and acted more slowly on mature biofilms. No biofilm overgrowth was observed when applying the two phage species combination, over a 48 h period of imaging by confocal microscopy. Genetic mutants resistant to each phage arose at a frequency of 10^−5^ to 10^−6^ per generation, and most *P. LS1*-resistant mutants were sensitized to the *B. LUZ24* phage. The combination of the selected phages has promising properties that are relevant in the framework of phage therapy.

## INTRODUCTION

*Pseudomonas aeruginosa* is a ubiquitous Gram-negative bacterium found in freshwater and terrestrial environments. It is also an opportunistic pathogen in humans, causing bloodstream, urinary tract, or burn wounds infections, as well as airway infections in cystic fibrosis patients or patients under mechanical ventilation (1, 2). Among its many virulence factors, the three polysaccharides alginate, Psl, and Pel are particularly important, as they permit the establishment of chronic biofilm infections and immune evasion (3), which is the main cause of ventilator-associated pneumonia (VAP). Some clinical isolates (such as PAO1) will build up their biofilm mostly on Psl, whereas others (such as PAK1) mostly on Pel, and a third category on a mix of both Pel and Psl (3, 4). In all cases, however, Psl seems needed for the attachment step of the biofilm, and monoclonal antibodies targeting Psl have been tested for adjunctive use with antibiotics to treat biofilms (5). Psl polysaccharide is a neutral polymer composed of repeating pentasaccharides of D-mannose, D-glucose, and L-rhamnose, with a cell-associated and a cell-free form (6). How the Psl polysaccharide is attached to the bacterial surface remains unknown at present. Regulation of its synthesis is multi-layered and relies in particular on the intracellular levels of di-cyclic-GMP (7–9). Interestingly, even during planktonic growth in liquid medium, Psl-dependent aggregates tend to form in early exponential growth (OD 0.05–0.2) and to dissociate later on (10).

Additionally, *P. aeruginosa* belongs to the ESKAPE group of pathogens for which multidrug resistance is an increasing threat. Indeed, *P. aeruginosa* infections are becoming more difficult to treat, with fewer antibacterial drugs available, resulting in higher mortality and morbidity rates (11). There is therefore an urgent need for novel therapeutic strategies, such as phage therapy (12, 13).

Similar to the outcome of antibiotic treatments, placing bacterial populations under phage attack will lead to the selection of clones resistant to phages. This has been observed under *in vitro* settings, as well as in the mouse gut (14, 15), and in some cases of patients treated with phage therapy over long periods of time (16). In short-term *in vitro* experiments with *P. aeruginosa*, bacterial resistance to phage was found to consist of acquiring either CRISPR-Cas spacers against the phage or surface receptor mutations (17–19).

To maintain phage infectivity regardless of bacterial evolution, the use of different phages—both in terms of taxonomy and bacterial receptor targeted—will be a good strategy (20). This should limit the rise of phage-resistant bacteria in the patient undergoing phage therapy, as there is a lower likelihood of bacterial mutants arising with modification to two different receptors. Similarly, strains (or populations of bacteria) acquiring spacers against different phages are less likely to emerge. Moreover, it was demonstrated that the CRISPR-Cas system is associated with an infection-induced fitness cost: although mutants acquiring spacers are initially selected for, the receptor mutants are selected in the long term (21).

For *P. aeruginosa*, most characterized phages use a limited set of receptors, such as the lipopolysaccharide (LPS) or the type IV pili (19, 22, 23). In addition, the phage OMKO1 (φKZ-like) was found to bind another receptor, OprM, an outer membrane porin. Interestingly, this porin is also used by two multidrug efflux pumps, MexAB and MexXY (24). Some of these receptors can also be considered virulence, or fitness factors, in which case phage-resistant clones have fitness trade-offs, compromising their survival under *in vivo* conditions (25, 26). A case of phage-resistant mutations affecting efflux pumps was also found to resensitize the strain to antibiotics (24). The use of phages targeting virulence factors as receptors could make phage therapy doubly effective: in addition to killing a large part of the bacterial population, it may drive multidrug-resistant bacterial pathogens to evolve toward phage resistance at the cost of decreased fitness and ability to survive in the host. It is thus important to identify the bacterial receptors of virulent phages that might be used in phage therapy.

A large screening of *P. aeruginosa* phages active against an international panel of *P. aeruginosa* strains (27) led us to select four phages with complementary spectra of action, with the goal of use in phage therapy. Two of these phages were myoviruses belonging to the species *Pbunavirus LS1* (below, these two phages will be collectively referred to as *LS1* phages), and two were podoviruses belonging to the species *Bruynoghevirus LUZ24* (*LUZ24* phages below). Whereas the primary receptor of the *LS1* phage vB_PaeM_PA01_Ab27 (accession NC_026586, designated Ab27 below) had already been characterized as being the O-antigen part of the LPS (28), no information was available at the onset of this work on the receptor used by *LUZ24* phages (29). We therefore undertook this characterization, which led us to uncover a new receptor for *P. aeruginosa* phages, the Psl polysaccharide, confirming the conclusion reached recently for another *LUZ24* phage (30). We also found that phages from both species were able to degrade biofilms, with different kinetics and final efficiency depending on the biofilm support.

## MATERIALS AND METHODS

### Bacterial strains, plasmids, and culture conditions

Bacterial strains and plasmids used in this study are listed in. Table S1. Unless indicated, *Escherichia coli* and *P. aeruginosa* strains were grown at 37°C, 200 rpm in Luria Bertani Broth containing 5 g/L NaCl (Formedium), which will be designated below as Lennox. The minimal medium used was “Modified MOPS Medium” with 20 mM acetate, designated below MMMa (31). Compared to the usual MOPS medium (32), CaCl_2_ and FeCl_2_ were added. Sputum Medium was prepared as described in reference 33. To grow strains in partial aerobiosis, the Lennox medium was covered with 2 mL of sterile paraffin over the 10 mL culture and incubated without agitation in a 50 mL sterile plastic tube. Moreover, KNO_3_ was added at 100 mM to favor *P. aeruginosa* anaerobic growth (34). For *E. coli* strains, gentamicin (Gm) was added when required at 30 µg/mL. For *P. aeruginosa* strains, antibiotics and chemicals were added at the following concentrations: isopropyl β-D-1-thiogalactopyranoside (IPTG): 0.5 mM, and Gm: 10 µg/mL for pSV35 derivatives, and 50 µg/mL for pSEVA629M containing strains. Bacterial strains were stored at −80°C in Lennox or MMMa, with 20% glycerol.

### Construction of strain PAO1-3Δ

Strain PAO1_OR (accession LN871187) contains two prophages, Pf4 and Pf6. Unless stated otherwise, a PAO1_OR derivative in which these prophages were inactivated was used throughout this work and named PAO1-3Δ. Starting from PAO1_OR, deletion of Pf4, of the *rep* gene of Pf6, and of *toxA* was performed successively by allelic exchange, using plasmids pMB01, pMB03, and pMB06, respectively (Table S1). Each deletion involved three steps (unless specified otherwise). In the first step, a non-replicative pEX18ApGW derivative in which the region to be deleted was replaced by an *aac1* gene, and flanked by ~300 bp regions homologous to the bacterial chromosome, was introduced by single crossing-over into the chromosome with a gentamicin resistance selection. In a second step, deletion of the plasmid while retaining the recombination construct was selected by plating bacteria on sucrose and gentamicin, as the presence of the *sacB* gene on the pEX18ApGW plasmid renders the strain sensitive to sucrose. In a third step, the *aac1* gene, which is flanked by FRT sequences, was excised with plasmid pFlp2. For the last step of the *toxA* gene deletion, to avoid introducing a third FRT scar, no *aac1* gene was placed between the regions flanking the deletion, and vector pEXG2 was used instead of pEX18ApGW. The construction involved only the first two steps, and *toxA* mutants were screened by PCR. The final strain PAO1-3Δ was completely sequenced and assembled. The three constructed deletions were as expected. Two additional mutations were generated during the construction steps: a 1 bp deletion within the gene *PAO1OR3357* encoding a hypothetical protein, and a CAC deletion within a run of 15 CAC repeats of the *czcD_2* gene, coding for a cadmium, cobalt, and zinc antiporter.

### Plasmid constructions

Primers used in this study are listed in Table S2. All PCRs were done using Phusion High Fidelity DNA Polymerase (New England BioLabs). All plasmid cloning steps were performed in *E. coli* strain JM105. To generate the fragments needed for the cloning of pMB01, pMB03, and pMB06, each of the two or three PCR fragments to be assembled was first amplified separately, and then a splicing overlap extension PCR was allowed to join the fragments together. Final plasmid construction integrity was verified by Sanger sequencing.

To complement *pslA* mutants, the *pslA* ORF and its 20 preceding nucleotides, including the RBS, were PCR amplified on DNA from PAO1-3Δ, using primers pslA_5′ and pslA_3′ (Table S2), and the PCR product was digested by *Kpn*I and *Eco*RI and cloned into the *Kpn*I/*Eco*RI digested plasmid pPSV35, downstream of the Plac promoter, to generate pPSV35-*pslA*. Integrity of the cloned open reading frame (ORF) was verified by Sanger sequencing. The same approach was used to clone the *pslD* ORF in pPSV35 (primers pslD_5′ and 3′) and generated plasmid pPSV35-*pslD* (Table S2).

Most plasmids were introduced into PAO1 strains by conjugation. For this, the plasmid was first transformed into strain β2163, which contains in its chromosome all genes needed for the RP4 conjugation process. Its growth depends on the addition of 0.3 mM of diaminopurine. Next, the plasmids, containing the RP4 oriT transfer origin, were transferred into the recipient strain by conjugation, as described in reference 35. The pGEX2 derivative, as well as the pSEVA629M plasmid, was introduced into *P. aeruginosa* strains by electroporation.

### Phages

**Fig 1:**
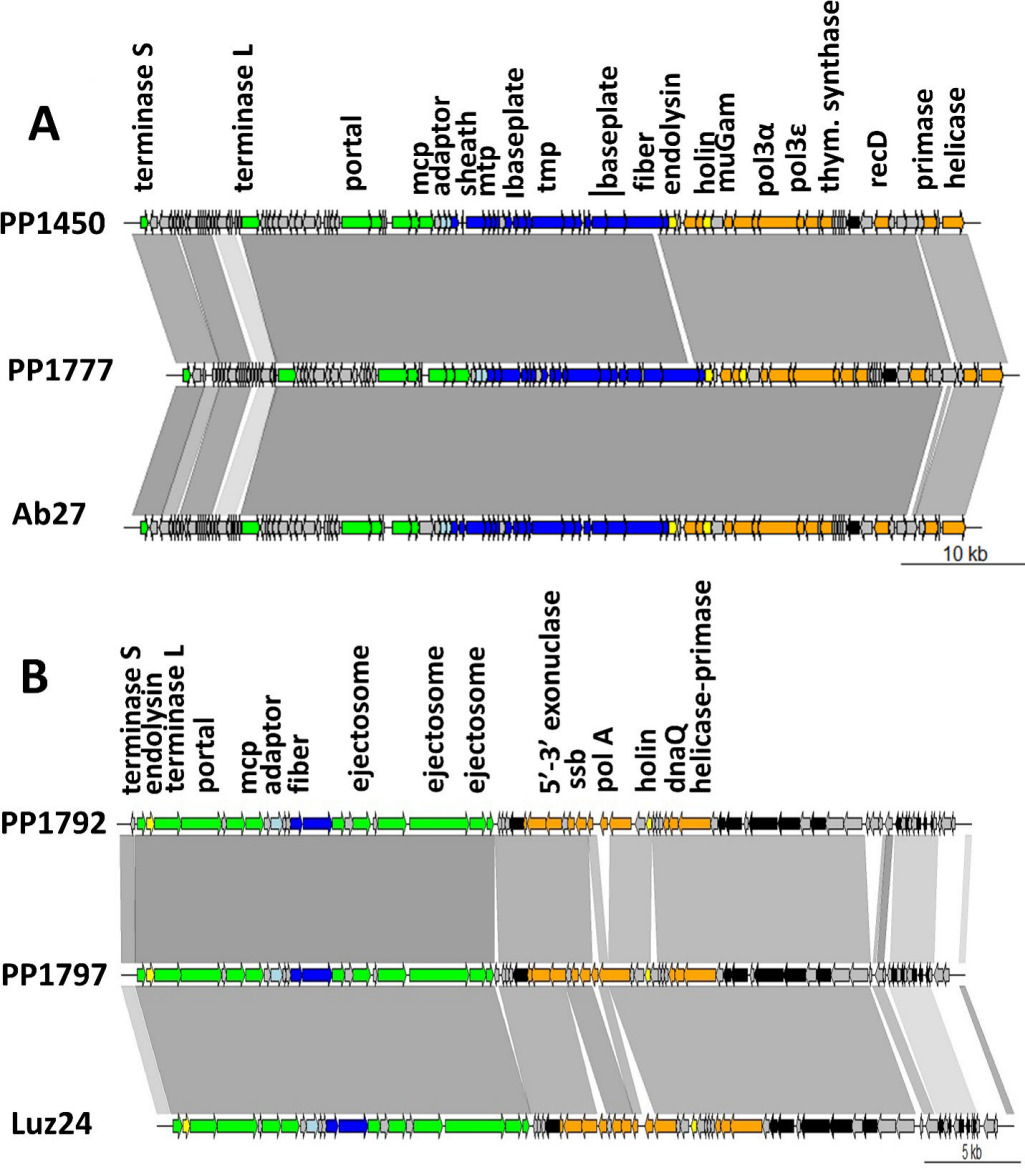
(**A**) Genomic alignment of phages PP1450 and PP1777 with Ab27 (orientation and first nt of PP1450 and PP1777 were adapted to align with Ab27). (**B**) Genomic alignment of phages PP1792 and PP1797 with Luz24 (orientation and first nt of PP1792 and PP1797 were adapted to align with Luz24). Genes are colored according to their functional modules. Green, capsid and encapsidation; light blue, connector; dark blue, tail; orange, DNA replication; black, auxiliary metabolic genes; gray, hypothetical proteins. The gradient of gray between the phage maps represents the BLASTn identity value (90% to 100% nt identity range).

The four phages used in this study, PP1450 (species *P. LS1*), PP1777 (species *P. LS1*), PP1792 (species *B. LUZ24*), and PP1797 (species *B. LUZ24*), were isolated from wastewater samples. Their genomes were sequenced (accession numbers LV539990 [PP1450], LZ998055 [PP1777], LZ998056 [PP1792], and LZ998057 [PP1797]) and functionally annotated. For this, coding sequences were first predicted with RAST (36) using genetic code 11, virus option, and RASTtk pipeline. Different tools were used to predict protein function, and then annotated by consensus. Tools included (i) PsiBLAST search against the Conserved Domain Database (37) with an E-value cutoff of 10^−5^, (ii) remote homology search of structural genes against the Virfam database (38), (iii) global remote homology search using HHblits against the PHROGs database (39), and (iv) remote homology search with the HHPred interface against the PDB, Pfam-A_v35, and UniProt-SwissProt_viral70_3_3_Nov_2021 databases (40). In all cases, biological function predictions were retained if HHsearch probability was above 95%. The genome alignments of Fig. 1 were generated with BLASTn and drawn using GenoplotR (41).

### Growth monitoring of bacteria during phage infection

Culture growth in 96-well plates was monitored as follows: overnight cultures were diluted to ~10^6^ colony-forming units (CFU)/mL in fresh medium. Bacteria and phages were added to each well in a final volume of 150 µL, exposing bacteria (~10^5^ CFU/mL) to phages (~10^5^ PFU/mL) at a multiplicity of infection (MOI) of 1. Plates were incubated at 37°C while shaking. OD_600 nm_ was measured every hour for 24 h using a MultiSkan Go or a LogPhase 600 plate reader.

For large volume cultures, overnight cultures were diluted to OD_600_ = 0.05 in fresh medium. At OD_600_ = 0.5, corresponding to ~10^8^ CFU/mL, 5 mL of bacterial cultures were infected with each phage (either alone or in combination) at an MOI of 1, incubated with no agitation for 10 min at 37°C, then diluted 10-fold in 50 mL of pre-warmed Lennox medium, and incubated at 37°C for several hours with shaking, in a 250 mL flask. The OD_600_ was followed over time. To compare growth kinetics, the area under the curves (AUC) was computed by the trapezoid method, and the relative AUC of a given phage treatment is its ratio over the non-infected AUC, in each experiment. The activity of two phages was defined as additive if the relative AUC of the combination was equal to the product of each phage’s relative AUC. If surface reduction was at least twice more than this product, the effect was considered to be synergistic.

### Fluctuation assay with the Luria Delbrück test

Fluctuation assays were performed as initially described by Luria and Delbrück (42), either in Lennox or MMMa. Overnight cultures were diluted 100-fold and incubated at 37°C with shaking. At OD_600_ = 0.5, the culture was diluted to 10^6^ CFU/mL, divided into 90 wells of the 96-well plate (100 µL), and mixed with 10^8^ PFU (100 µL) of phages . The six remaining wells contained the growth medium (two wells); the growth medium with phage and no bacteria (two wells); and the growth medium with the tested bacterial strain and no phage. The plate and its plastic cover were then wrapped in parafilm to avoid evaporation and incubated at 37°C, 200 rpm for 24 h for Lennox growth and 36 h for MMMa growth. After this incubation time, the number K of clear cultures, i.e., in which all bacteria were killed (meaning that in this well, no resistant mutant was present at the time of infection), was monitored. If K was between 20 and 80, we considered that the fraction K/90 was an estimation of the first term of the Poisson law with mean λ (K/90 = e^-λ^), describing the occurrence of a mutation with low frequency in the population. Mutation frequency *f* was then obtained by dividing λ by the average number of bacteria per well N, at infection time [*f* = −Ln (K/90)/N].

### Selection of bacterial strain resistant to phage, sequencing, and mutation analysis

Bacterial mutants were recovered either at the end of the small volume fluctuation assays or after overnight growth of large batch cultures in phage infection experiments. For large batch cultures, the culture the next morning was serially diluted 10^6^ and 10^7^-fold and plated. For fluctuation test assays, three wells with bacterial growth per experiment were chosen randomly and streaked on agar plates containing the same growth medium. Each mutant was streaked three times on either Lennox or MMMa to eliminate residual phages. After the purification process, mutants were grown overnight in Lennox or MMMa at 37°C, 200 rpm, and stored at −80°C with 20% glycerol.

DNA from mutants resistant to *LS1* or *LUZ24* phages was extracted, and either entirely sequenced (Illumina HiSeq) or sequenced only for the genes of interest by Sanger technology. Shotgun sequencing was performed on an Illumina HiSeq platform (Eurofins Genomics, 2 × 125 bp, depth: 9 million reads). Mutations were identified on reads using Breseq (43), with PAO1-3∆ as the reference genome.

### Spot test and efficiency of plating assay

The plating efficiency of the four phages on *P. aeruginosa* mutant strains was determined by spot assay: 10 µL of a serial 100-fold dilution of a phage preparation was spotted on double-layer agar plates inoculated with each bacterial host. The number of plaques observed after overnight incubation was compared to the phage titre on its reference strain: PAO1-3∆ for PP1450 and PP1777; NAR71 for PP1792 and PP1797.

### Adsorption assay

Bacteriophage adsorption assay was performed as previously described (44). Briefly, overnight bacterial cultures incubated at 37°C were diluted in fresh medium at an OD_600_ of 0.05 and incubated at 37°C until reaching OD_600_ = 0.5. Phages were then added at MOI 0.01, and the adsorption proceeded at 37°C for 10 min without shaking. Then, samples were collected and either centrifuged at 10,000 × *g* for 5 min or treated with 50 µL of chloroform (both methods gave comparable results), and supernatants were titrated by spot test. As a reference, a negative control, with phage only and no bacteria, was performed and titrated.

### Surface Psl extraction and Psl immunoblots

Surface Psl extraction was performed following the protocol described in (6). Briefly, for all samples, an equal number of bacteria, equivalent to 5 mL of a culture at OD 2, was centrifuged for 10 min at 10,000 × *g*. For cultures grown in rich medium, to remove residual Psl from supernatants, bacteria were washed twice with fresh Lennox, by centrifugation. The clean pellet was then resuspended in 200 µL EDTA 0.5 M, boiled at 95°C for 20 min. The bacterial debris was then removed by centrifugation for 5 min at 16,000 × *g*, and the supernatant was treated with proteinase K at 0.2 mg/mL and incubated for 1 h at 60°C. Finally, proteinase K was inactivated by incubation of the samples at 80°C for 30 min. Psl extracts were kept at 4°C for a maximum of 1 week before testing. No treatment was applied to recover Psl from culture supernatants. Bacteria were simply pelleted by centrifugation (5,000 × *g*, 5 min), and the supernatant was filtered with 0.2 µm filters and kept at 4°C.

To quantify Psl, 5 µL of samples were deposited on a 0.45 µm nitrocellulose membrane (Amersham Protran ref 10600003) and let dry. Membranes were then incubated in TBST buffer (50 mM Tris pH 7.5, 150 mM NaCl, 0.1% Tween 20) complemented with 1% fat-free powder milk (Regilait Bio) for 1 h in a rotating shaker at room temperature, in a volume of 5 mL. Then, human anti-Psl monoclonal antibody (Creative Biolabs. Ref MRO-160MZ) was added at a 1/5,000 dilution, and incubation was continued for 1 h. After three short rinses of the membrane with TBST, the secondary antibody (Goat-anti-human IgG antibody coupled to HRP, Sigma, Ref AP112P) was applied in 5 mL TBST buffer, at a 1/5,000 dilution, and incubated for 1 h under the same shaking and temperature conditions. After three short rinses of the membrane with TBST, the revealing reagent (Clarity Western ECL substrate, BioRad ref 170-5060) was poured on the membrane, which was imaged 1 min later, in a Chemidoc apparatus (BioRad). Image analysis was performed with the ImageLab software of BioRad, using non-saturated images and volume counts adjusted with local background. A standard curve using serial dilutions of the most concentrated Psl sample showed a linear signal over a 27-fold dilution range (Fig. S3, panel B).

### Biofilm quantification on a medical device

The intubation device (Hexamed, reference 44295LCM/93350LCM) used to grow biofilms was prepared as follows: balloons, bevel connections, and the small injection tube were removed; the remaining tubing was cut into 1 cm long cylinders. Each cylinder was then cut into four longitudinal sections, and each piece was placed in a well of a 96 U-shaped well plate (Thermofisher Scientific, reference 167425). Wells were seeded with 150 µL of an overnight culture of the tested bacterial strain diluted 1/1,000 in Lennox. Plates were covered and incubated for 7 h without shaking at 30°C. Each intubation piece was then washed by three successive immersions in phosphate-buffered saline (PBS) and placed in a clean 96-well plate. Phages were added at a final concentration of 10^9^ PFU/mL in 150 µL Lennox (estimated bacterial amount, 5 × 10^5^ CFU per tubing piece, MOI ~100), covered and incubated for 17 h at 30°C without shaking.

Crystal violet coloration was performed as follows: each intubation piece was washed by three successive immersions in PBS and placed in a 96-deep well plate (VWR, reference 732-3325). Wells were containing 500 µL of 0.01% crystal violet solution, and pieces were incubated for 30 min at RT without shaking. The excess of crystal violet was removed by three successive immersions in PBS to allow biofilm visualization. Crystal violet was then dissolved in 500 µL of 33% acetic acid for 10 min with shaking and quantified by OD at 590 nm. After the subtraction of the blank (33% acetic acid), the test OD was normalized by the intubation piece weight. Each experiment included triplicates, and a point on the graph represents the average of the three phage-treated replicates divided by the average of the untreated condition.

### Quantification of biofilm growth in 96-well plates by confocal microscopy

Strain MB198, a PAO1_OR derivative in which plasmid pSEVA627M (45) was introduced, was always propagated in Lennox complemented with 50 µg/mL gentamicin. Its constitutive GFP expression permitted the following of living bacteria within the biofilm. To detect dead bacteria, propidium iodide (PI, Invitrogen) was used (16–80 µM final concentration). Prior to microscopy analysis, a growing or a mature biofilm was grown for 6 and 16 h, respectively, at 37°C, under static conditions, in a 96-well plate (microscopic-grade Phenoplate-96, Perkin Elmer). To inoculate each well, an overnight culture of MB198 was diluted to an OD of 0.05 in Lennox, and a 200 µL volume was applied to the well and incubated for 1 h at 37°C statically, to let bacteria adhere. Next, the 200 µL was removed and replaced with fresh Lennox and placed back in the incubator. After biofilm growth, phages were added at 5 × 10^6^ (estimated bacterial count at phage addition, 10^7^ CFU per well; MOI ~0.5) and 5 × 10^8^ PFU (estimated bacterial count, 2 × 10^8^ CFU per well, MOI ~2.5) for the short and long pre-incubations, respectively, in a 50 µL volume of SM buffer (50 mM Tris pH 7.5, 100 mM NaCl, 10 mM MgSO_4_) mixed with PI; uninfected wells received 50 µL of SM buffer mixed with PI, and the plate was placed under the microscope. The phage addition constituted time 0 of the experiments. Confocal imaging was performed using an HCS-SP8 Leica Confocal laser scanning microscope (LEICA Microsystems, Germany) at the INRAE MIMA2 microscopic platform (doi.org/10.15454/1.5572348210007727E12). A 3D time-lapse acquisition was initiated, capturing image stacks every hour for a total duration of 48 h. Each image stack was acquired with a 1 µm z-step interval. Images (512 × 512 pixels covering a 184.52 µm × 184.52 µm area) were captured at a 600 Hz frequency using a 63× water objective lens with a numerical aperture of 1.2. For GFP detection, excitation was set at 488 nm, and emission was collected in the 500–550 nm range. For PI, excitation was also at 488 nm, with red fluorescence captured in the 600–750 nm range. 2D projections were generated using Imaris 9.3.1 software (Bitplane, Zurich, Switzerland). Quantitative biofilm parameters, including biovolume, thickness, and roughness, were extracted using BiofilmQ software (46).

## RESULTS

### The four virulent phages infecting *P. aeruginosa* selected for phage therapy belong to two phage species, *Pbunavirus LS1* and *Bruynoghevirus LUZ24*

Four *P. aeruginosa* infecting phages were selected for therapeutic use, based on their efficient lytic capacities and good coverage of an international reference panel of *P. aeruginosa* strains (27). Their genomes were sequenced and compared to phage genomes at NCBI (BLASTn). Two of them, PP1450 and PP1777, were closely related to the 66 kb Ab27 genome (28), a myovirus belonging to the *Pbunavirus LS1* species. They shared with Ab27 98.11% and 97.41% nucleotide identity, respectively, over 97% of their genome length (Fig. 1A). We concluded they belonged to the *LS1* species, like Ab27. The other two phages, PP1792 and PP1797, were closely related to the 45 kb Luz24 genome (29), a podovirus of the *Bruynoghevirus LUZ24* species. They shared with Luz24 97.02% and 96,59% nucleotide identity, respectively, over 94% of their genome length (Fig. 1B). They belonged, therefore, to the *LUZ24* species.

Two phages per species were chosen for therapeutic use because, despite their partial overlap, they had interesting complementary host ranges. Variable regions were therefore searched for within putative receptor binding proteins. Among the large genomic changes visible between the two *LS1* genomes, there was a short region within a tail fibre gene that may correspond to the receptor binding protein. No similar large modification was observed in the tail nor the fiber gene of the two *LUZ24* genomes (dark blue genes in Fig. 1), but some amino-acid polymorphisms were detected in the fiber gene product (Fig. S1), which may also be at the root of the differing host ranges of PP1792 and PP1797.

None of these four phages encoded an integrase gene, confirming their virulent lifestyle. No toxins or antibiotic resistance genes were detected in these genomes, making them suitable for therapeutic use.

### Mutants resistant to *LS1* phages arise at a frequency of 6 to 7 × 10^−6^ per generation

To study phage host interactions, a PAO1_OR derivative deleted of its prophages was constructed (see Materials and Methods for strain construction) and designated PAO1-3Δ. To estimate the frequency at which mutants resistant to each *LS1* phage arise, fluctuation assays were performed. For this, PP1450 or PP1777 were propagated at high MOI on a given concentration of PAO1-3Δ bacteria, in Lennox medium in 90 replicate cultures, and the number of cultures in which mutants arose allowed the determination of mutation frequency, as described in the Materials and Methods. This frequency was 6.1 (±4.1) × 10^−6^ per generation for PP1450, and 7.0 (±1.7) × 10^−6^ for PP1777.

### The receptor of the two *LS1* phages is the O-antigen of the lipopolysaccharide

To characterize the phage-resistant mutants obtained, wells in which bacterial growth emerged were selected, and mutants were isolated by three successive streaks on Lennox medium. For five independent mutants (three resistant to PP1450, two resistant to PP1777), complete genome sequencing was performed, and reads were then analyzed to search for mutations (Table 1).

**TABLE 1:** Mutations detected in the genomes of PP1450- or PP1777-resistant mutants

Strain name	Selection conditions	Genes mutated/ lost	Detected mutations	Protein modification
MB79	Fluctuation assay with PP1777	*wzy1*	A:7→6 (nt 136)	T54^*a*^
MB75^*b*^	Fluctuation assay with PP1450	*wzy2*	A:7→8 (nt 136)	R74^*a*^
MB81	Fluctuation assay with PP1450	*wzy2*	A:7→8 (nt 136)	R74^*a*^
MB76	Fluctuation assay with PP1450	*wzy3*	G:6→7 (nt 625)	T276^*a*^
MB78	Fluctuation assay with PP1777	Numerous, including *galU*	Δ 329,240 bp	NR^*c*^

^*a*^ Indicates the stop codon.

^*b*^ Mutant MB75 had an additional mutation in the *gloA2* gene (R50H).

^*c*^ "NR“ indicates not relevant.

Four of these five mutants had a mutation in the *wzy* gene, which encodes a polymerase involved in the biosynthesis of the LPS O-antigen chain, resulting in truncated proteins (Table 1). The last mutant had a large deletion including the *galU* gene, which is also needed for the biosynthesis of LPS. Its defect results in the absence of the O-antigen chain and a defective outer core. This type of large deletion has been observed repeatedly with the PAO1 strain, including among mutants resistant to phage infection (19, 47). The two *LS1* phages had different plating efficiencies on the mutants: PP1450 was more affected than PP1777 on *wzy* mutants, whereas conversely, PP1777 was more affected than PP1450 on the large deletion mutant MB78 (Table S3).

**Fig 2:**
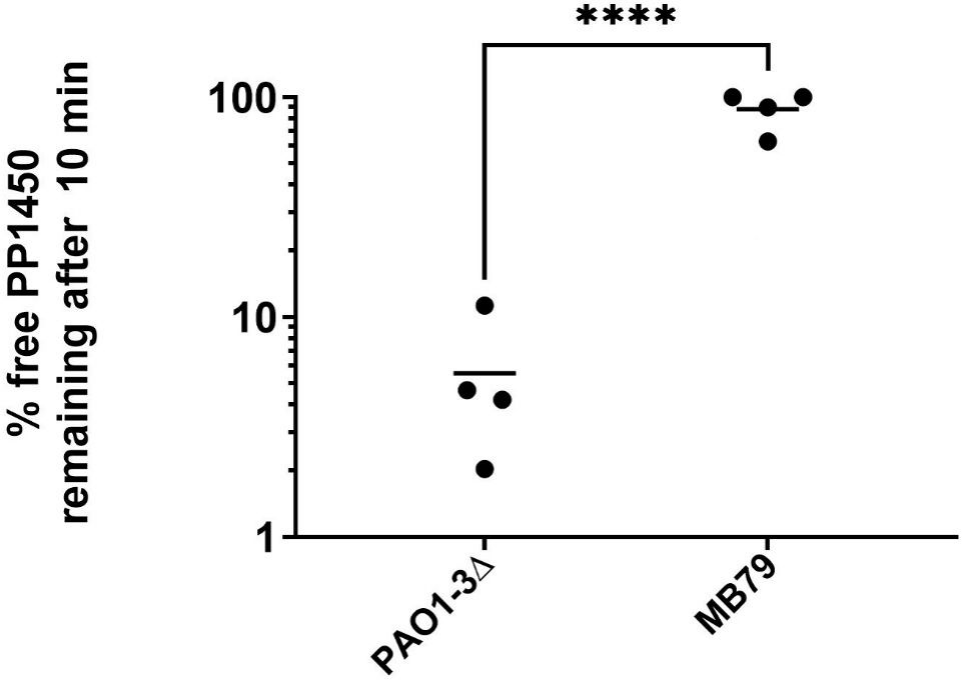
Percent of free phage remaining in the supernatant after adsorption of PP1450 for 10 min at 37°C in Lennox medium (**** = unpaired *t*-test, *P* < 0.0001).

Mutant strain MB79 (Table 1), with a deletion of an A in a run of 7 A resulting in a truncated protein of 54 amino acids (mutation *wzy1*), was kept for further study. We verified that compared to PAO1-3Δ, the adsorption of PP1450 on MB79 was significantly decreased (Fig. 2: 5.5 [±2]% free phage remaining for PAO1-3Δ and 88.2 [±8.7]% for MB79; *P*-value <0.0005). Sequencing of the *wzy* gene PCR product in six additional resistant clones also revealed mutations in this gene, so that in total, among 11 sequenced mutants, 10 were in the *wzy* gene, and one was a deletion including *galU*. Altogether, these results suggest that, in agreement with results obtained for Ab27 (28), the O-antigen chain LPS is the receptor of phages PP1450 and PP1777.

### *LS1* and *LUZ24* phage infections affect PAO1-3Δ growth differently, depending on growth conditions

**Fig 3:**
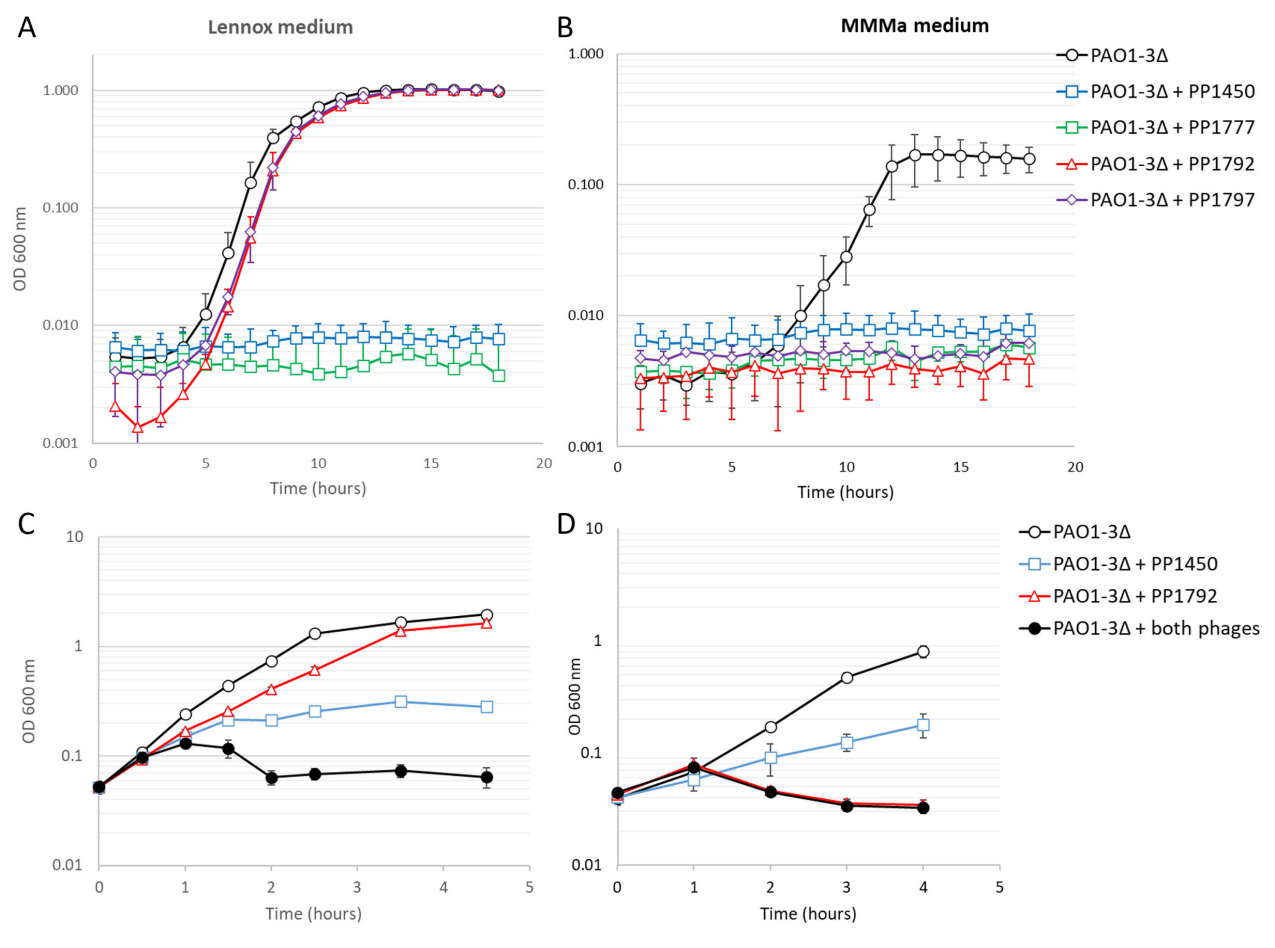
(A and B) Growth kinetics of strain PAO1-3Δ in the presence or absence of a single phage (MOI = 1) in 96-well plates in Lennox medium (**A**) or MMMa (**B**). (C and D) PAO1-3Δ growth kinetics of 50 mL aerated cultures in Lennox medium (**C**) or MMMa (**D**), in the presence or absence of PP1450 (MOI = 1), PP1792 (MOI = 1) or both (MOI = 1 for each phage). Average and standard deviation of three experiments per condition are shown.

The two *LUZ24* phages were able to multiply on strain PAO1-3Δ, but not sufficiently to generate visible growth inhibition in 96-well plates (Fig. 3A) nor in large cultures (Fig. 3C), and they usually did not form plaques on this medium. Therefore, we could not select any mutants resistant to *LUZ24* phages in this growth medium. We searched for conditions in which growth inhibition would be completer and found that in minimal medium containing acetate as a carbon source (MMMa), the PAO1-3Δ culture was fully cleared by the *LUZ24* phages (Fig. 3B). In comparison, the two *LS1* phages were as active in rich and minimal medium (Fig. 3A and B).

To further explore this improved phage activity and analyze the effect of a phage combination, the same experiment was repeated in large and aerated cultures (50 mL), using *LS1* phage PP1450, *LUZ24* phage PP1792, or a combination of both (Fig. 3C and D; Table S4). In rich medium (bacterial generation time 31 ± 2 min), the phage combination had a larger effect than PP1450 alone, since the AUC of the PP1450 infected culture was 17 (±2)% of the un-infected culture, and it dropped to 6 (±1)% for the combination PP1450 and PP1792 (Table S4). The relative AUC of the PP1792 infection was 77 (±2)% of the uninfected culture, so that an additive effect of the two phages was expected to be of 13 (±2)% relative AUC (Table S4). The more pronounced inhibition suggests some synergistic effect of the two phages. It indicates that PP1792 was not completely inactive in rich medium. In minimal medium (bacterial generation time 51 ± 4 min), the reverse effect was observed, with PP1792 performing full inhibition, whereas PP1450 activity was partial, and the combination did not generate increased inhibition compared to PP1792 alone. With a different phage combination (PP1777 and PP1792), growth inhibition by the *LS1* phage PP1777 was more pronounced than with PP1450 in aerated Lennox, and addition of the *LUZ24* phage did not accentuate the growth inhibition observed by PP1450 alone (Fig. S2C).

To investigate phage multiplication in growth media more closely resembling the conditions of *P. aeruginosa* infections in human tissues were tested: sputum medium (a medium mimicking the human lung sputum, bacterial generation time 61 ± 24 min), and growth in partial anaerobic conditions (static Lennox supplemented with potassium nitrate, and covered with paraffin, bacterial generation time 74 ± 3 min) (Fig. S2A and B). In these conditions, the lytic effect of *LUZ24* phage PP1792 was again more pronounced than that of *LS1* phage PP1450. The two-phage combination had a stronger effect in sputum medium, but not in a partial anaerobic condition. The lytic activity of the *LUZ24* phage was superior in slow-growth conditions compared to rich medium. It should be noted, however, that following overnight incubation, bacterial growth resumed in all cases, including with the phage combination.

We conclude that the efficiency of growth inhibition of the four phages under study depends on growth conditions, and that in rich medium, the assembly of the *LS1* with the *LUZ24* phage has a synergistic effect (Fig. 3C), whereas in sputum medium, the effect is additive (Fig. S3).

### The polysaccharide Psl is the receptor of the two *LUZ24* phages

**Fig 4:**
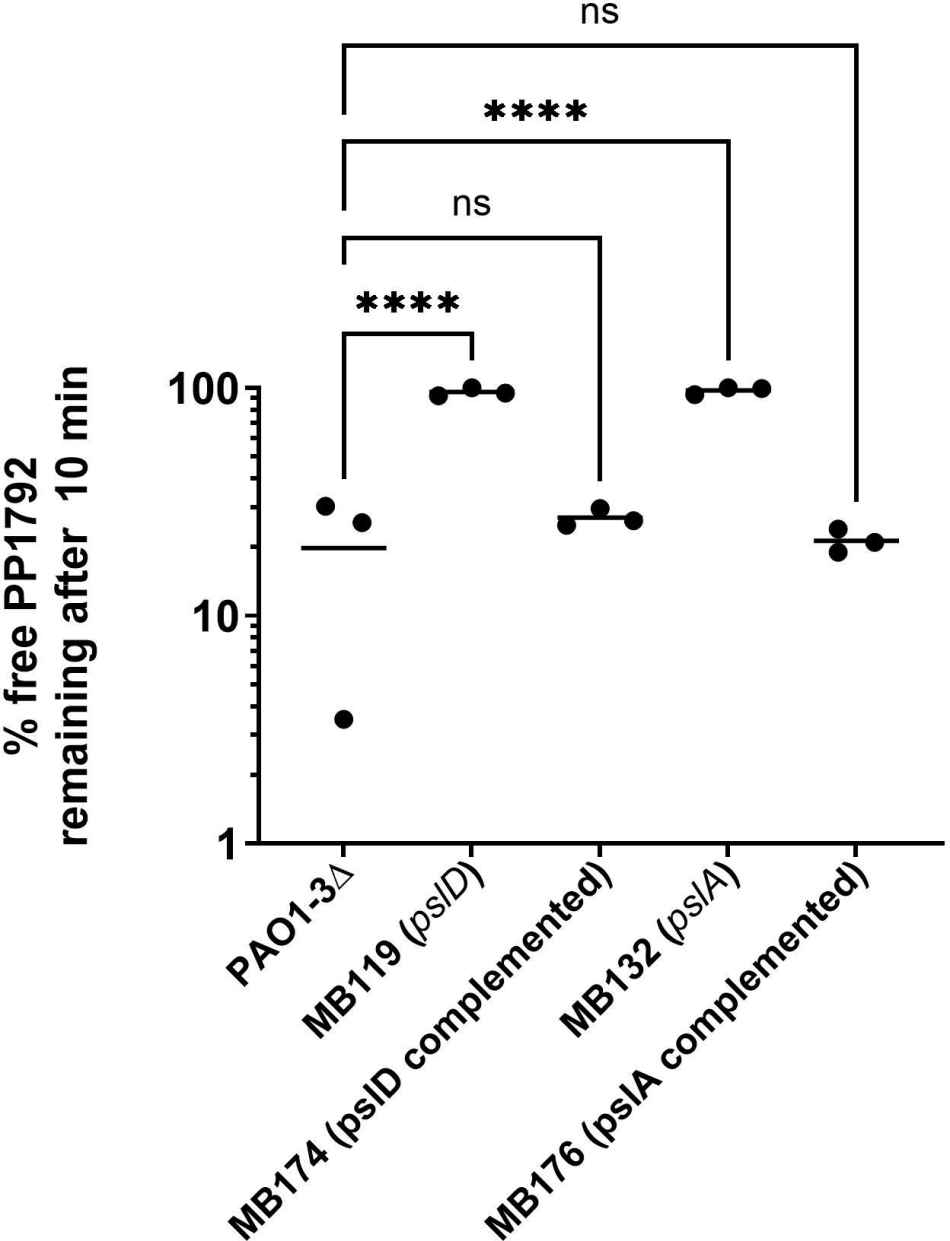
Percent of free phage remaining in the supernatant after adsorption of PP1792 for 10 min at 37°C in MMMa medium. One-way analysis of variance (ANOVA) comparisons to PAO1-3Δ, only significant values are shown (****P < 0.001).

Once conditions favorable to *LUZ24* infection were established, growth in MMMa medium was used to study the bacterial receptor of these phages. To determine at which frequency mutants resistant to *LUZ24* emerged, fluctuation assays were conducted as described above for *LS1* phages, except that Lennox was replaced by MMMa, and the plates were read after 48 h of growth at 37°C. Mutation frequency was 1.8 (±1.2) × 10^−5^ per generation for PP1792, and 3.8 (±3.6) × 10^−5^ per generation for PP1797. Four mutants resistant to PP1792 were isolated, purified, and confirmed to be PP1792-resistant. To determine whether these mutants were affected at the adsorption step, adsorption efficiency after 10 min of incubation at 37°C was measured. Three out of the four mutants abolished PP1792 adsorption (Fig. 4). Whole-genome sequencing of these mutants revealed a mutation in the *psl* operon (Table 2): strains MB132 and MB133 had the same 13 nt deletion in *pslA*, named *pslA1*, while strain MB119 had a 17 nt deletion in *pslD*, named *pslD1*. The *pslA* gene encodes a glycosyl-transferase, while *pslD* encodes a transporter component, and both gene deletions were shown to abolish Psl production at the bacterial surface (6). Two additional mutants obtained from the fluctuation assay with PP1797 were also sequenced and revealed a new mutation in *pslA*, as well as a mutation in *pslH* (Table 2).

**TABLE 2:** Mutations present in PAO1-3Δ adsorption mutants resistant to *LUZ24* phages

Strains name	Selection conditions	Genes mutated	Detected mutations	Protein modification
MB132	Fluctuation assay in MMMa with PP1792	*pslA1*	Δ13 bp (928–940 nt/1,437 nt)	Y327^*a*^
MB133	Fluctuation assay in MMMa with PP1792	*pslA1*	Δ13 bp (928–940 nt/1,437 nt)	Y327^*a*^
MB119	Infection in batch MMMa culture with PP1792	*pslD1*	Δ17 bp (133–149 nt /795 nt)	N80^*a*^
MB153	Fluctuation assay in MMMa with PP1797	*pslA2*	Δ4 bp (812–815/1,437 nt)	R280^*a*^
MB154	Fluctuation assay in MMMa with PP1797	*pslH1*	Δ1 bp (700/1,209 nt)	A258^*a*^

^*a*^ Stop codon.

To confirm that these mutations were sufficient to suppress adsorption, a complementation assay was conducted. Strains MB132 (*pslA1*) and MB119 (*pslD1*) were transformed with plasmid pPSV35-*pslA* expressing *pslA* and plasmid pPSV35-*pslD* expressing *pslD*, respectively. These complemented strains were grown in MMMa supplemented with gentamycin and IPTG and tested for their capacity to adsorb PP1792. Phage PP1792 absorption to the *pslA* and the *pslD* complemented strains (21.3 [±2.5]% and 26.9 [±2.4]% of free phages remaining after 10 min of adsorption) was comparable to the PAO1-3Δ strain (19.8 [±14.3]%; Fig. 4). In addition, PP1792 and PP1797 lysed the complemented strains with an efficiency of plating similar to the PAO1-3Δ strain, while being unable to infect the *psl* mutant strains *pslA1* and *pslD1* (Table S5). Taken together, these data show that the receptor for the *LUZ24* phages PP1792 and PP1797 is the Psl polysaccharide.

### Psl binds more efficiently to the bacterial surface in MMMa than in Lennox during exponential growth

**Fig 5:**
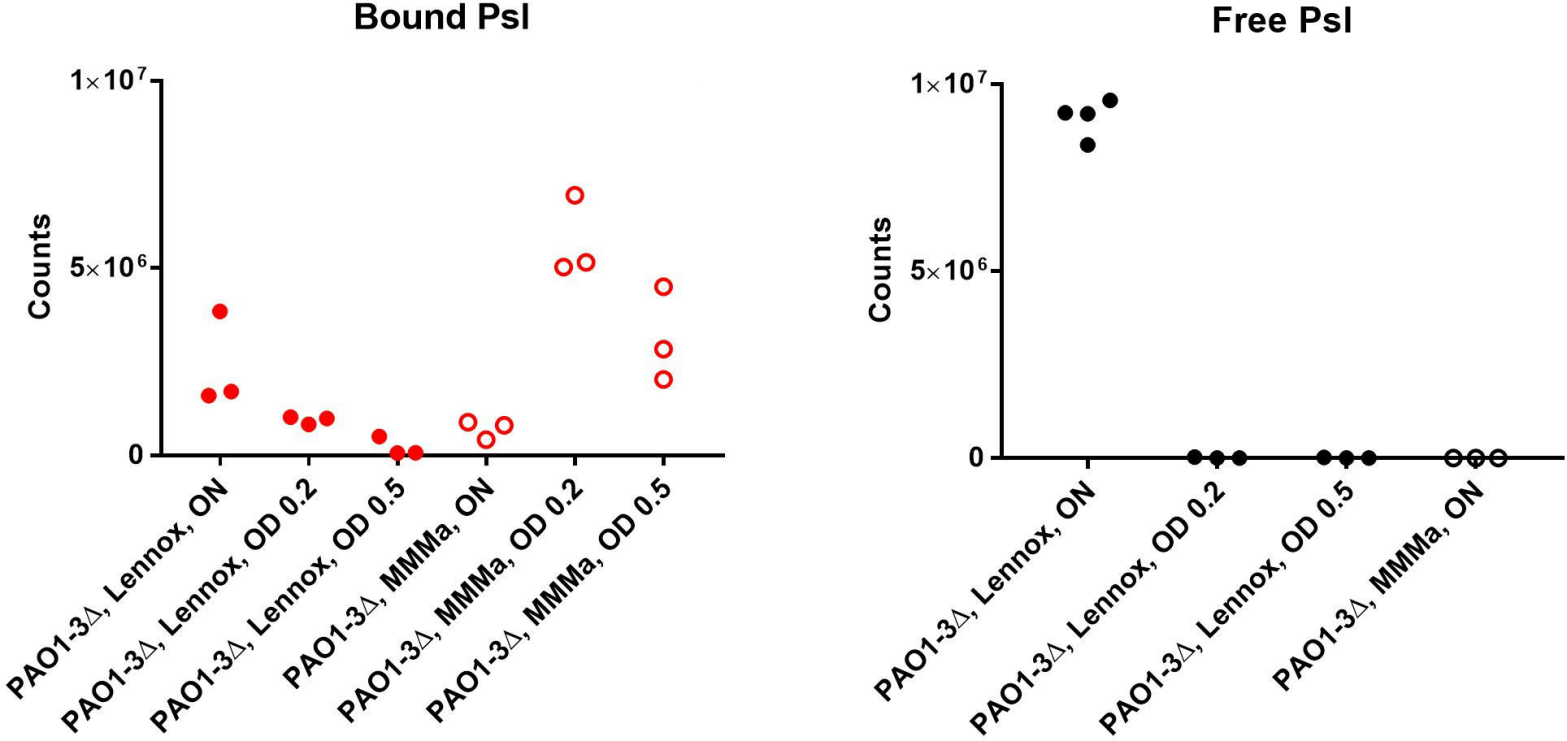
Psl amounts quantified from western blots. Bacteria collected during exponential growth at OD 0.2 and 0.5 or from overnight (ON) cultures were used to extract surface-bound Psl from equivalent amounts of washed pellets (left side, “bound Psl”), and these extracts were then deposited on a membrane and treated for immunodetection as described in the Materials and Methods. In parallel, 5 µL of supernatants from the same cultures (right side, “free Psl”) were directly deposited on the same membranes.

The Psl polysaccharide exists both in an attached form to the bacterial surface, as well as in a free form in bacterial supernatant, and in rich medium, the attached form is transiently present at the onset of the exponential phase (10). We therefore investigated whether the reason for the better infection of *LUZ24* phages in MMMa could be an increased fraction of bound Psl under this growth condition. Surface-bound, as well as free Psl, were collected from PAO1-3Δ cultures grown in Lennox or MMMa, at various optical densities, and immunodetected with Psl monoclonal antibodies (Fig. 5). We found that in MMMa, all Psl was present on the bacterial surface, and no Psl was present in the supernatant of an overnight culture. In contrast, in rich medium, surface-bound Psl was not abundant during exponential growth (16% and 7%, relative to the bound Psl amounts measured for bacteria grown in MMMa medium, for OD 0.2 and 0.5, respectively). It became abundant at the surface of overnight cultures (3.4-fold more bound Psl, relative to overnight MMMa cultures) and accumulated massively in supernatants of overnight cultures (Fig. 5; Fig. S3).

We conclude that bacteria grown in MMMa had larger amounts of bound Psl, compared to those grown in Lennox medium, especially in the exponential phase, a context favorable to phage *LUZ24* multiplication.

### The Psl-binding *LUZ24* phage PP1792 is more efficient at biofilm removal on medical endotracheal tubes than the *LS1* phage PP1777

**Fig 6:**
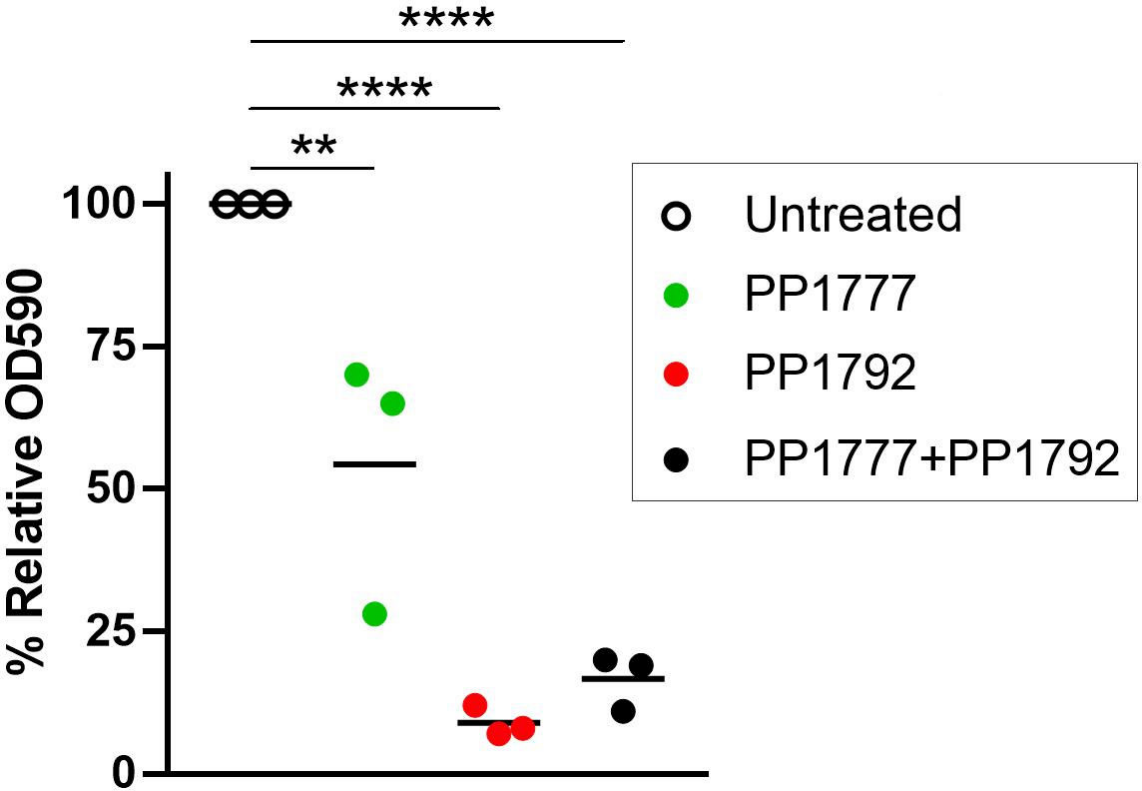
Biofilm quantification by crystal violet coloration, expressed as a percent of the untreated condition (gray dots). One-way ANOVA tests, **P < 0.01; ****P < 0.001.

The Psl exopolysaccharide is involved in biofilm formation, which can develop on the endotracheal tube of ventilated patients and cause VAP. To evaluate the relevance of phage therapy in such a setting, biofilms of PAO1-3Δ were grown on pieces of endotracheal tubes (Fig. S4). After 7 h of biofilm formation at 30°C, 1.5 × 10^8^ PFU of phage were added individually or in combination (when in combination, 1.5 × 10^8^ PFU of each phage was added, so 3 × 10^8^ PFU phages in total) and incubated for 17 h. The biofilm was quantified using crystal violet coloration (Fig. 6). On the PAO1-3Δ biofilm, both phages significantly decreased the amount of biofilm, especially *LUZ24* phage PP1792, which decreased the amount of staining by a factor of 10. The combination of the two phages significantly decreased the biofilm to a level equal to that of *LUZ24* phage PP1792.

### Both *LUZ24* and *LS1* phages are able to kill sessile bacteria within biofilms

To explore the kinetics of phage-mediated biofilm degradation, biofilms were grown in 96-well plates, and a confocal microscopy set-up was used to track the biofilm content of living bacteria over 48 h after phage addition. Instead of using our reference PAO1-3Δ strain, its more natural ancestor, PAO1_OR, hosting two filamentous prophages, was chosen. These prophages contribute to biofilm growth and stability over time (48). The strain also contained plasmid pSEVA627m, a low-copy-number plasmid expressing the GFP gene constitutively under a strong promoter (45), allowing for the detection of living bacteria.

After 6 h of biofilm formation at 37°C, *LUZ24* phage PP1792 (5 × 10^6^ PFU), *LS1* phage PP1450 (5 × 10^6^ PFU), or a combination of both (2.5 × 10^6^ of each phage, ~5 × 10^7^ total PFU) were added (MOI ~0.5), and biofilm behavior was monitored. Phage addition prevented biofilm growth during the subsequent 16 h. Interestingly, bacterial overgrowth later took place for each single phage addition, but not for the phage combination (Fig. S5). This indicated that phages are able to attack bacteria during the first stage of biofilm attachment, and if given in combination, to prevent overgrowth for at least 48 h.

**Fig 7:**
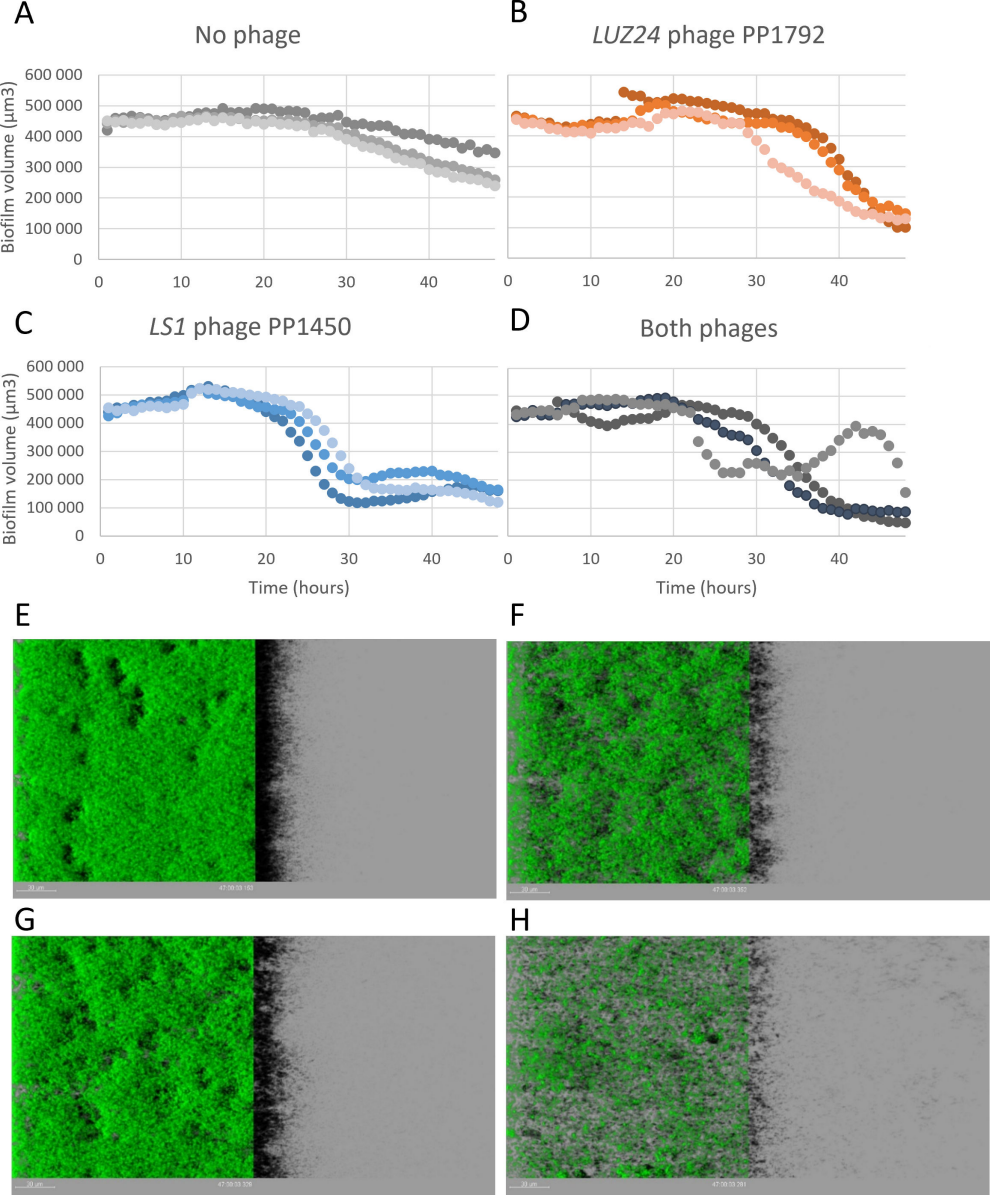
Quantification of living cell volumes (µm^3^, y-axis) within a PAO1_OR biofilm, as a function of time (hours, x-axis), on a 16 h biofilm treated with no phage (**A and E**), *LUZ24* PP1792 (**B and F**), *LS1* PP1450 (**C and G**), or both phages (**D and H**). Confocal imaging was performed every hour over 48 h. Panels E–G show the last image of one movie by treatment.

We next investigated whether phages can also attack a biofilm grown to maturation for 16 h at 37°C in Lennox in a 96-well plate. After 16 h of static growth, 5 × 10^8^ PFU of a single phage, or 2.5 × 10^8^ PFU of each phage in the two-phage combination, were added (MOI ~2.5), the plate was placed under the microscope at 37°C, and imaging was initiated. Three technical repeats were placed on each plate, and three biological repeats were performed. One repeat is shown in Fig. 7 (repeats 2 and 3 are shown in Fig. S6 and S7). The last image of a representative movie of each phage treatment for each biological replicate is also shown (Fig. 7E through H).

The number of living cells in the untreated biofilm could be maintained stable over the 48 h post-imaging in one biological replicate (Fig. S6A), but it decreased after 28 h (Fig. 7A) and 23 h (Fig. S7A) of imaging in the other two. The occasional late decrease in viable cells (slope of an average 8,000 to 14,000 µm^3^/h for the two replicates in which decay took place) was likely due to nutrient depletion over the long incubation time (64 h in total). Addition of the *LS1* phage PP1450 resulted in a steep decrease of viable cells in the biofilms (22,000 ± 10,000 µm^3^/h), which always took place earlier than the natural decay, starting between 8 and 21 h post-phage addition. The *LUZ24* phage PP1792 infection took place 3 to 10 h later than the *LS1* attack. In the absence of natural decay (Fig. S6), biofilm degradation started 11 h post-addition (and 3 h after *LS1*), whereas in the two replicates with natural decay, a faster degradation started 2 h after the beginning of this decay. In all cases, however, a steep slope (21,000 ± 5,000 µm^3^/h) was observed, suggesting phage infection was taking place.

The combination of the two phages did not manifest an additive effect in kinetics parameters (time of inflexion, slope) compared to infections with each phage alone and was most similar to the *LS1* PP1450 kinetics parameters (Fig. 7D; Fig. S6D and S7D; Table S6). On average, at the end point of all kinetics, including the phage combination, the volume of living cells was 3.9 ± 2.2-fold less than in the t48 time point of the untreated biofilms (Fig. 7H). This killing activity was slightly, but not significantly, superior to that for each phage alone: the PP1792 treatment led to an average 2 (±1)-fold final reduction in living cell volume, and the PP1450 effect was 1.8 (±1)-fold. This tendency of a better final killing activity of the combination is probably due to the absence of regrowth, contrary to what is observed for 5 of the 17 single phage experiments (see Fig. S6B, C, and S7B). Interestingly, one of the eight biofilms treated with the phage combination also exhibited some regrowth, but it was followed by a second degradation step (Fig. 7D). Mature biofilms proved much more difficult to degrade with the tested phages, compared to the initial stage of biofilm formation (phage addition after 6–7 h of biofilm growth), but some signs of phage attack could be observed.

### Searching for mutants resistant to phages with different receptors: *LS1*-resistant strains are sensitized to *LUZ24* infection in rich medium

To explore the capacity of our phage combination to prevent bacterial regrowth of resistant mutants, various phage applications to planktonic bacteria were tested. First, the ability of each phage species to grow on receptor mutants that prevent the growth of the other phage species was tested. *LS1* phage growth was effective on *pslA* and *pslH* mutants, and *LUZ24* phage growth was effective as well on the *wzy1* mutant strain MB79, on MMMa. Interestingly, on this MB79 mutant strain, *LUZ24* growth became effective even in rich medium (Fig. S8; Table S3).

**Fig 8:**
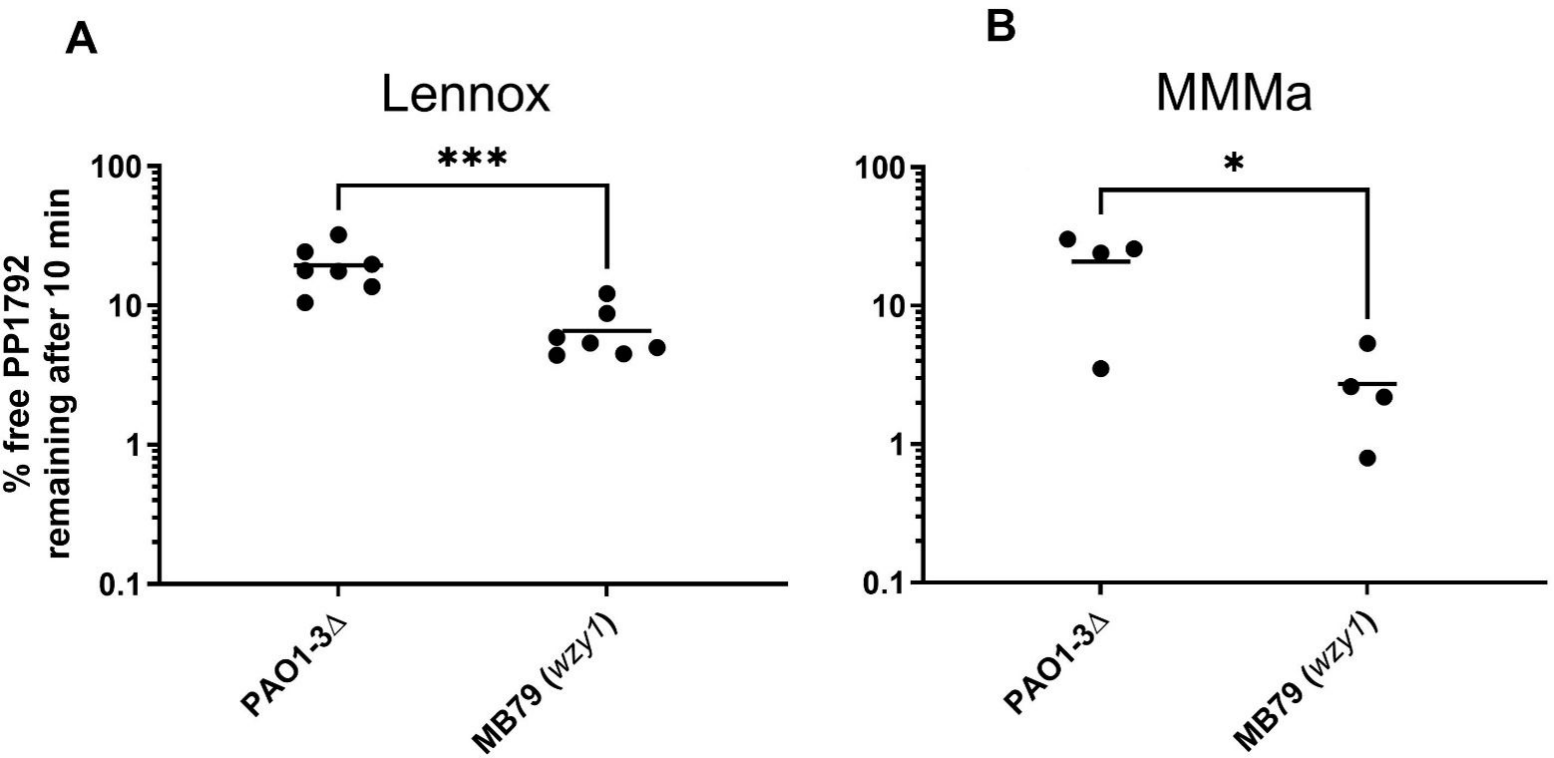
Percent of free phage remaining in the supernatant after adsorption of PP1792 for 10 min at 37°C on PAO1-3Δ and its *wzy1* derivative MB79, in rich Lennox medium (**A**) and in MMMa minimal medium (**B**). Unpaired *t*-test comparisons (Student test, ***P = 0.1%, *P = 2%).

In search of the reason for such an improvement, it was first hypothesised whether surface-bound Psl was increased for this mutant in rich medium. This was not the case (Fig. S9). Next, whether adsorption efficiency was improved was investigated, and it was found that *LUZ24* phage PP1792 adsorption was significantly more efficient on strain MB79 (*wzy1*), compared to PAO1-3Δ in Lennox (Fig. 8A). The fact that phage adsorption is more efficient, while receptor concentration is not increased, suggests that the lack of *LUZ24* infection in rich medium is due to a shielding effect of the O-antigen chain of the LPS.

Interestingly, in minimal medium, *LUZ24* phages were able to infect the PAO1-3Δ, despite the presence of the O-antigen chain (Fig. 3B and D), suggesting that this shielding effect was overcome. This may be due to the more elevated amounts of surface-bound Psl in this medium, permitting it to counterbalance the O-antigen shielding effect. The two effects are probably additive, as it was observed that *LUZ24* phage PP1792 adsorption was also more efficient on the *wzy1* mutant than on PAO1-3Δ, in minimal medium (Fig. 8B). This shielding effect was also visible by eye: in the presence of the O-antigen chain, *LUZ24* phages formed turbid plaques on MMMa as well as on Lennox (on this medium, some plaques were sporadically detected), whereas they formed clear plaques on the *wzy1* mutant strain MB79 (both in Lennox and MMMa). An interesting consequence of these analyses is that the O-antigen mutants that are often selected by *LS1* phage infection are sensitized to *LUZ24* phage infections in rich medium.

To further our understanding of the emergence of phage-resistant mutants, a set of five independent mutants was analyzed that emerged from PAO1-3Δ co-infected with *LS1* phage PP1777 and *LUZ24* phage PP1792, upon growth in Lennox liquid cultures. These mutants were resistant to all four phages (Table S7). Whole-genome sequencing revealed for three of them a mutation or a deletion involving the *galU* gene, whose function is needed for both LPS and Psl synthesis. The fourth mutant was in gene *PAO1OR_5105* of unannotated function. InterPro annotates the corresponding protein (accession K9HUG4) as a putative glycosyl transferase involved in LPS synthesis. The last mutant affected the *ygiF* gene, encoding a putative inorganic triphosphatase. We speculate that YgiF influences the synthesis of LPS and Psl. By hydrolyzing inorganic triphosphate, YgiF was proposed to help maintain calcium homeostasis (49), which is crucial for regulating polysaccharide production. Calcium ions play a key role in biofilm formation, and disruptions in calcium signaling can impact the synthesis of LPS and Psl, particularly in species like *P. aeruginosa* (50, 51).

Finally, to mimic successive infection by an *LS1* phage followed by a *LUZ24* phage, resistant mutants were isolated starting from the *wzy1* mutant strain MB79, infecting it with *LUZ24* phage PP1792 and analyzing overgrowth in liquid Lennox medium. Five mutants were purified and analyzed for their sensitivity profile to the four phages (Table S8). Most strains (4/5) had the same phenotype; they were totally resistant to the two *LUZ24* phages and remained resistant to *LS1* phage PP1450, but became partially sensitive to *LS1* phage PP1777. The last mutant, MB99, was different: it was totally resistant to the two *LS1* phages and to the *LUZ24* phage PP1797, but remained sensitive to PP1792. These mutants were whole-genome sequenced. Four of them had a mutation in the *fklB* gene, coding for a peptidyl-prolyl cis-trans isomerase (PPiase) of the FkpA family. FkpA is a periplasmic protein having both a chaperone and a PPiase function in *Escherichia coli* (52). The last mutant MB99 had a different mutation in the *rocS1* gene (Table S7). Adsorption assays of the *LUZ24* phage PP1792 were conducted on strains MB91 and MB92, which are two different *wzy fklB* double mutants, as well as on MB99, the *wzy rocS1* mutant (Fig. S10). They revealed that the *fklB* mutation did not affect the adsorption step, while the *rocS1* mutation did.

Interestingly, none of these five mutants had the expected mutation in a *psl* gene, suggesting that the double *wzy psl* mutant might not be viable, at least in rich medium. The search for resistant mutants was therefore repeated in MMMa, and four resistant clones were purified and analyzed for their sensitivity profiles. Two had a typical *galU* profile (resistant to all phages), and the last two were resistant to the two *LUZ24* phages, but sensitive to the two *LS1* phages (MB187 and MB189, Table S8). These two strains were sequenced and had three mutations: the initial *wzy1* mutation of strain MB79, a mutation in either *pslA* or *pslH,* and a last mutation in *wbpL*, adding a G in a run of 9Gs. This gene codes for a glycosyl transferase needed for the synthesis of both A and B chains of the O-antigen.

## DISCUSSION

This study investigated the phage-host relationships between a prophage-less derivative of the PAO1_OR strain of *P. aeruginosa*, PAO1-3Δ, and four phages selected for their capacity to kill collectively a wide range of the 43 strains of an international reference panel (27). Two of these phages belonged to the *Pbunavirus LS1* species, and their receptor was the O-antigen of LPS, as expected for this species. The two others belonged to the *Bruynoghevirus LUZ24* species, and for these phages, although previously characterized (29), the host receptor remained unknown. To find the receptor of the *LUZ24* phages PP1792 and PP1797, various bacterial growth conditions of PAO1-3Δ were tested. Whereas inefficient clearing of PAO1-3Δ cultures was observed in Lennox medium liquid culture, and only turbid spots at high phage concentrations were visible on Lennox agar plates, the phages inhibited growth in liquid in minimal medium MMMa and formed individual turbid plaques on this medium. In addition, *LUZ24* infection was more efficient upon bacterial growth in Sputum medium, as well as under partial anaerobic conditions (i.e., upon static growth with a paraffin top layer). These *LUZ24* phages should, therefore, be active when their host has a metabolism adapted to slow growth, such as inside human wounds or lungs (53).

Efficient *LUZ24* phage growth in MMMa allowed for the selection of bacterial mutants resistant to phage infection, and 5/6 of these were adsorption mutants. This permitted the discovery that *LUZ24* phages use the polysaccharide Psl to bind to the bacterial surface. Various *psl* mutants were obtained, with altered *pslA, pslH,* or *pslD* genes, all of which are necessary for the synthesis of the Psl polysaccharide (6). Recently, an independent study reached the same conclusion that Psl was the primary receptor of their *LUZ24* phages Clew1-Clew4, using a completely different approach (30). These phages were isolated thanks to a wastewater screening on a PAO1 *ΔpilA ΔfliF* mutant, in which the MS ring of the flagellum is affected. As here, Clew phages were unable to form plaques on PAO1 grown in rich medium, but they did so on *fliE*, *F,* or *G* mutants. Instead of changing the growth medium, a TnSeq screening with a Clew-infected *fliF* mutant strain allowed them to identify insertions in the *psl* operon as the most enriched ones. A set of genetic experiments also demonstrated that high c-di-GMP levels were necessary to increase Psl production and confer phage sensitivity. In another study, among bacterial mutants selected with *Bruynoghevirus* phage FJK, a *pslA* mutant was also obtained (20).

We also found that Psl was more abundant at the bacterial surface of exponentially growing cultures in minimal, MMMa medium, compared to rich, Lennox medium. This may explain why MMMa is a favorable context for phage growth, since the receptor is permanently available at the surface. Although overnight culture in rich medium contained large amounts of free Psl, this free Psl did not seem to compete for phage adsorption, since inoculation of rich medium with washed bacteria did not allow for improvement of bacterial growth inhibition in this medium.

Since Psl is the main polysaccharide forming the biofilm matrix in strain PAO1, the phage efficiencies on PAO1-3Δ biofilms formed during 7 h on the medical endotracheal tubes were evaluated. The biofilm matrix was significantly reduced by both *LS1* phage PP1777 and *LUZ24* phage PP1792, and to a greater extent by the latter, which decreased the matrix by a factor of 10 after a 16 h incubation. The activity of the phages on mature biofilms grown statically on 96-well plates (16 h of growth before infection) was also tested, using the parental, PAO1_OR strain, which was expected to be more difficult to eradicate, because its prophages contribute to biofilm stability over time (48). Moderate degradation occurred with both single phage treatments and started earlier with the *LS1* phage PP1450. Some regrowth was observed for four of the nine repeats with *LUZ24* phage PP1792, and one of the eight repeats with *LS1*. The combination of the two phages did not lead to a more rapid killing of the biofilm, compared to each phage alone, but interestingly, no visible overgrowth was observed for the eight replicates during the 48 h of the assay. This contrasts with an earlier report, where phages belonging to the same genera, even when applied in combination to a 24 h biofilm of PAO1, led to biofilm regrowth (measured with crystal violet) 8 h post-phage treatment (54). Kinetics of phage-mediated degradation of mature biofilms were surprisingly slow, an observation in line with a recent report showing that the peptidoglycan released upon phage-mediated biofilm degradation stimulates biofilm formation. This was shown for various bacterial species, including *P. aeruginosa* (55).

The complementary lytic characteristics of the *LS1* phages and the *LUZ24* phages could be beneficial in the frame of phage therapy. While the pair of *LS1* phages multiplied more efficiently on bacteria grown in aerated Lennox medium, the *LUZ24* phage pair had the opposite behavior, multiplying more efficiently on bacteria grown in minimal medium, as well as in Sputum medium or upon limited oxygen availability. On an infection site, there might be bacteria in different physiological states, and by combining these two phages, more bacteria should be killed, regardless of the state they are in.

As our two phage species targeted different receptors, their simultaneous application was expected to limit the emergence of resistant mutants (18, 19). However, *galU* mutants were resistant to both phage species, so the gain provided by the phage combination was not as high as expected. Nevertheless, in screenings, *galU* deletions arose at a lower frequency compared to *wzy* mutations (only 1/10 of *LS1* resistant mutants were *galU* deletions). Moreover, in plate assays, biofilm overgrowth did not appear over 48 h when a phage combination was applied to early (6 h) or late (16 h) biofilms, suggesting again that such mutants are rare. Unexpectedly, *wzy* mutants resistant to *LS1* phages (which occur 10 times more frequently than *galU* mutants) were sensitized to the *LUZ24* phages, in the rich medium where normally *LUZ24* phages are less active. This is an advantage of this phage combination.

The impact of the culture medium on phage-host interaction has not been thoroughly investigated until now, although it is starting to be considered. Lourenço et al. observed that in the gut microbiota, LPS host receptors on the *Escherichia coli* surface are not produced at sufficient levels to permit phage CLB-P1 multiplication (56). Moreover, the N4 phage receptor, an *E. coli* phage known for a long time, was also recently identified, thanks to the appropriate choice of culture conditions (57). This receptor is a novel surface glycan named NGR, the existence of which had escaped scrutiny until now. Interestingly, the synthesis of this receptor was dependent on the concentration of cyclic di-GMP in the cytoplasm, which itself was influenced by the levels of enzymes needed to synthesize or degrade cyclic di-GMP.

In one of the genetic screens aiming at characterizing bacterial mutant strains able to overcome phage infection, mutants were searched for that would arise from the successive application of an *LS1* phage (which generated mostly *wzy* mutants) followed by application of a *LUZ24* phage, in rich medium. The screen identified a single adsorption mutant, which had a *rocS1* mutation. This gene, also known as *sadS*, is a histidine kinase belonging to an intricate two-component system made of two sensors (*rocS1* and *rocS2*) and three regulators (*rocA1*, *rocA2,* and *rocR*). While RocA1 and RocA2 are HTH-containing DNA-binding proteins, the RocR regulator is different: it contains a diesterase domain and decreases the levels of cyclic di-GMP (58). By analogy with the N4 observations, we therefore hypothesize that a way to resist *LUZ24* phages is to modify the intracellular level of cyclic di-GMP, which might indirectly command the level of synthesis of the Psl polysaccharide, and thereby alter biofilm formation as well.

All other mutants characterized in this screen, mimicking successive application, were not adsorption mutants and had a mutated FklB. Two functions related to phage cycles are reported in the literature for FklB homologs. The FklB protein is homologous to the FkpA and SlyD proteins of *E. coli*. FkpA is a periplasmic protein necessary for HK97 phage injection (52). SlyD facilitates lysis during the lytic cycle of phage ΦX174 by stabilizing its protein E, an inhibitor of MraY (59). These two functions would enter into play after the initial adsorption step and are compatible with defects posterior to adsorption, as observed for the *fklB* mutants.

Overall, this genetic identification of the *LUZ24* phage receptor Psl allowed the uncovering of important characteristics of the infection process of this phage, namely its dependence on bacterial growth conditions. We also demonstrate here that the combination of the four phages selected for compassionate phage therapy treatments, two *LUZ24* and two *LS1* phages, has differing and complementary properties, making them an appropriate combination for treatments.

## SUPPLEMENTAL MATERIAL

Supplemental material aac.00579-25-s0001.pdf Fig. S1 to S10; Tables S1 to S8.

ASM does not own the copyrights to Supplemental Material that may be linked to, or accessed through, an article. The authors have granted ASM a non-exclusive, world-wide license to publish the Supplemental Material files. Please contact the corresponding author directly for reuse.
