# Supplementary material for: Complementary killing activities of Pbunavirus LS1 and Bruynoghevirus LUZ24 phages on planktonic and sessile Pseudomonas aeruginosa PAO1 derivatives
Source: Antimicrob Agents Chemother. 2025 Jul 23;69(9):e00579-25. doi: 10.1128/aac.00579-25 (PMC12406683; doi:10.1128/aac.00579-25)
Supplement: Supplemental material — Fig. S1 to S10; Tables S1 to S8. [file aac.00579-25-s0001.pdf]

Supplementary material

This file contains
10 supplemental figures, followed by
7 supplemental tables

—

Figure S1: polymorphism of the tail gene (ORF58) of phages PP1792, PP1797 and Luz24. Diverging amino-acids of phage PP1797, relative to PP1792 and Luz24 are shown in red.

```
1797     MGLEVATYINQLVPTNPTGSDLKSFGDDHLRLIKSAIKNTFPNISQAVTVTAAQLNAVAD  60
LUZ24    MGLEVATYINQLVPTNPTGSDLKSFGDDHLRLIKSAIKNTFPNISQAVTVTAAQLNAVAD  60
1792     MGLEVATYINQLVPTNPTGSDLKSFGDDHLRLIKSAIKNTFPNISQAVTVTAAQLNAVAD  60

1797     TTQYVKPGMVIMWAGSLAQIPAGWKLCNGVGTTSNGIPVPNLIGAFPWGIDGSSQAVGTR  120
LUZ24    TTQYVKPGMVIMWAGSLAQIPAGWKLCNGVGTTSNGIPVPNLIGAFPWGIDGSSQAVGTR  120
1792     TTQYVKPGMVIMWAGSLAQIPAGWKLCNGVGTTSNGVPVPNLIGAFPWGIDGSSQAVGTR  120

1797     GGSANIVWDGFTEGTALTLAQIPSHTHTWRTRGAAALVGSAGDSGALTGGSGNAANTNLE  180
LUZ24    GGSANIVWDGFTEGTALTLAQIPSHTHTWRSRGATTLTGSAGDSGALTGGSGNAANTNLE  180
1792     GGSANIVWDGFTEGTALTLAQIPSHTHTWRSRGATTLTGSAGDSGALTGGSGNAANTNLE  180

1797     TGPAGQGQTHNHAVKINMPLGNIPPFCSVFFIIKN  215
LUZ24    TGPAGQGQTHNHAVKINMPLGNIPPFCSVFFIIKN  215
1792     TGPAGQGQTHNHAVKINMPLGNIPPFCSVFFIIKN  215
```

Figure S2: *LS1* phage PP1450 and *LUZ24* phage PP1792 kinetics of lysis in 40 mL cultures of PAO1-3Δ either grown in Sputum medium (left panel) or in Partial anaerobiosis in Lennox (statically, with a paraffin layer to limit O2 diffusion inside the culture, middle panel). Right panel: lytic activities of *LS1* phage PP1777 and *LUZ24* phage PP1792 upon infection of PAO1-3Δ in 40 mL aerated Lennox cultures.

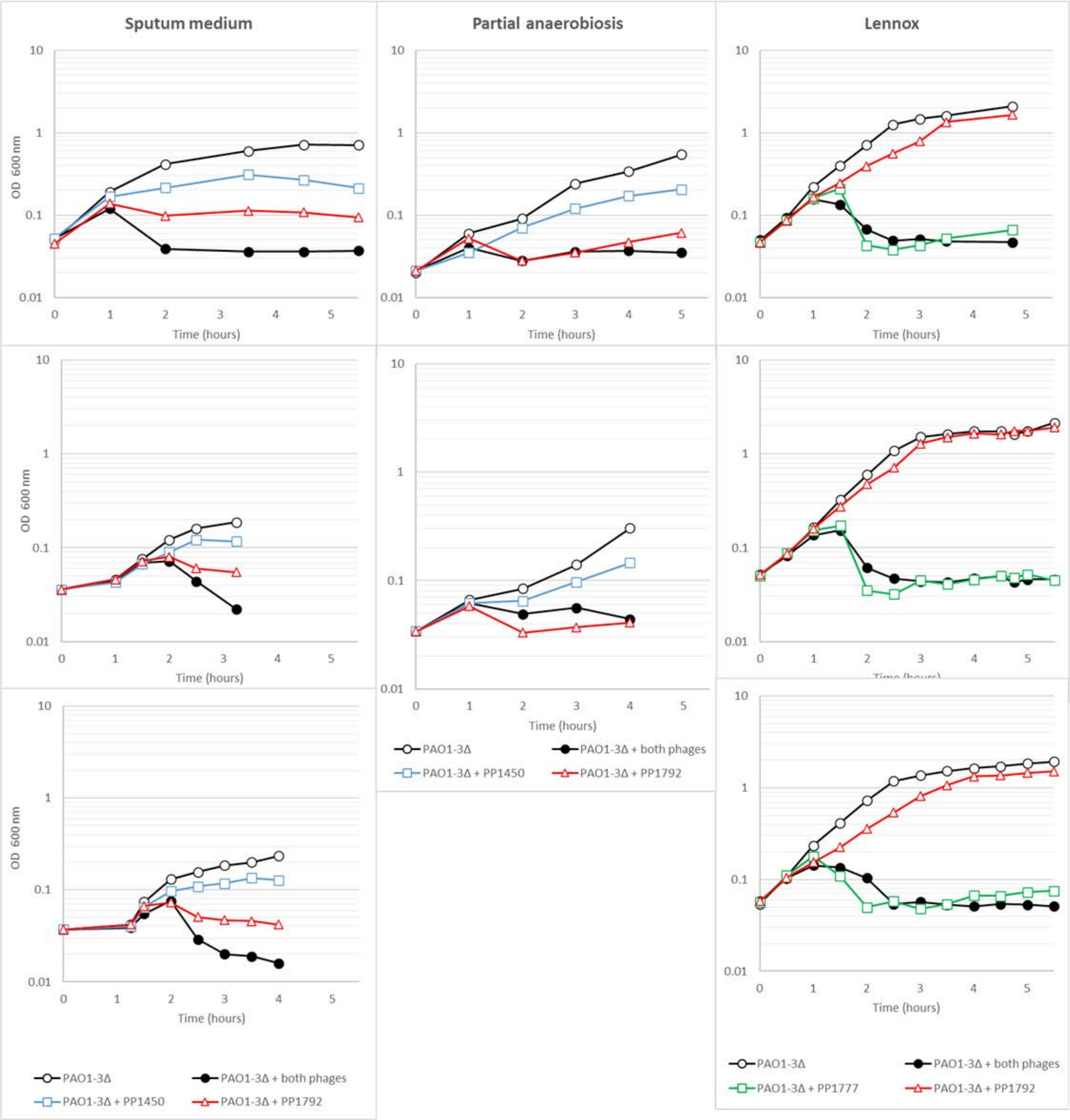

Figure S3: Immunoblot detection of Psl.

A*: Antibodies testing for the detection range*. Six serial 3-fold dilutions of the most concentrated Psl sample, which is a supernatant of the WT strain grown ON in Lennox. The top panel indicates the dilution factor of each sample deposited on the membrane. **B**: *Quantification of A*, dilutions below 0.04 are not detected, the background value corresponded to a volume of 30 000. All membranes for immunodetection contained this most concentrated sample as a reference, and images were recorded below the pixel saturation level of this sample. **C.** *Antibodies testing on negative controls*. Psl extractions were performed on strains MB119 (*pslD1* mutation) and MB132 (*pslA1* mutation) at the same OD tested for the WT strain, and samples were immunoblotted in parallel to the WT samples. Two independent extractions are shown. In addition, supernatants of the ON cultures of these two strains were also tested (one extraction shown). A signal close to the background was sometimes detected, but overall, the Psl antibodies were specific. **D**. *Psl detection with WT strain grown in MMMa*. Bound Psl from bacterial extractions (left, two independent extracts for each condition) and free Psl from supernatants (right, two independent extracts) are shown. **E**. *Psl detection with WT and MB79 strains grown in Lennox*. bound Psl from bacterial extractions (left, two independent extracts and blots) and free Psl from supernatants (right, two independent extracts and blots) are shown.

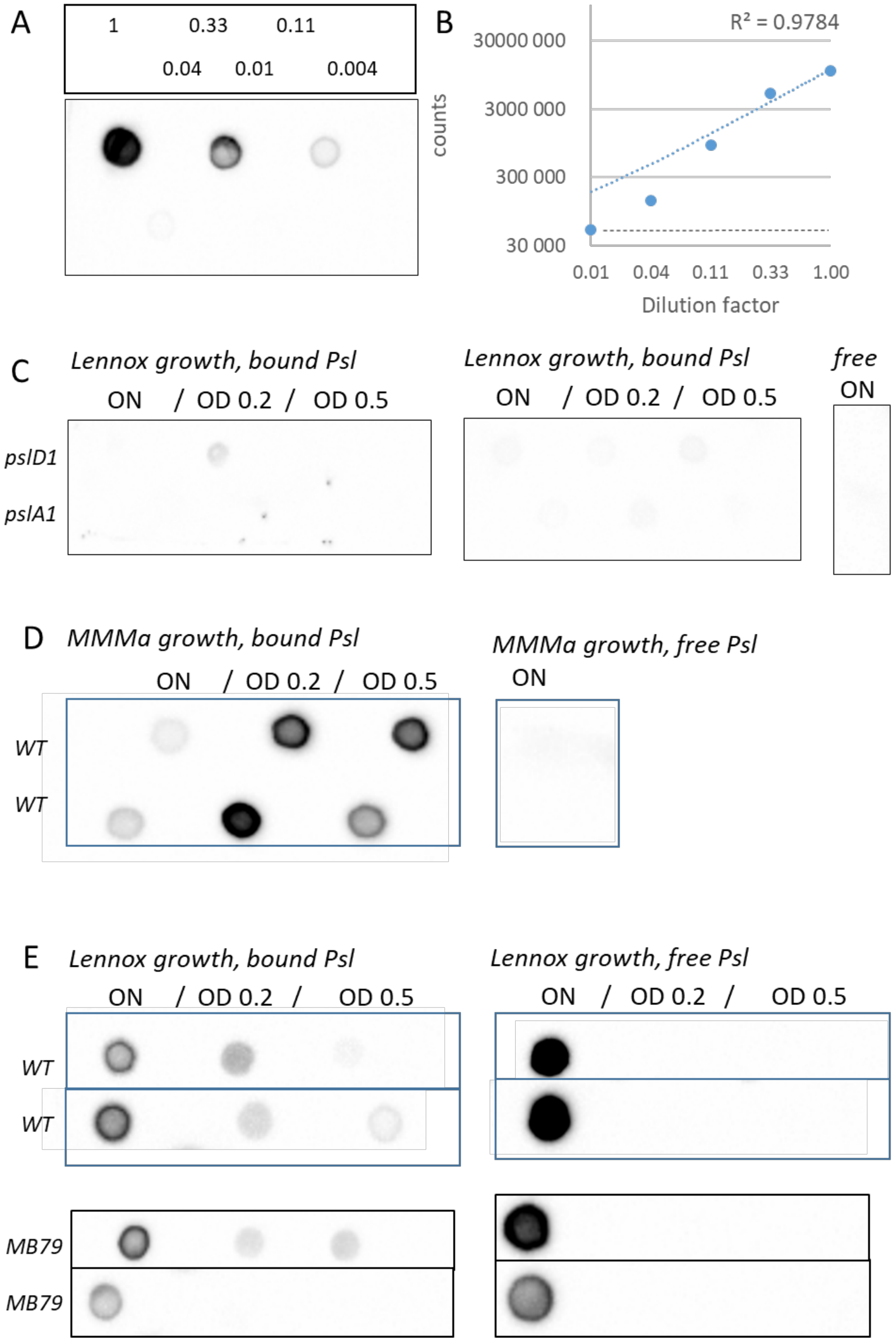
A
1
0.33
0.11
0.04
0.01
0.004
B
R² = 0.9784
30000 000
3000 000
300 000
30 000
counts
0.01
0.04
0.11
0.33
1.00
Dilution factor
C
Lennox growth, bound Psl
ON / OD 0.2 / OD 0.5
pslD1
pslA1
Lennox growth, bound Psl
ON / OD 0.2 / OD 0.5
free
ON
D
MMMa growth, bound Psl
ON / OD 0.2 / OD 0.5
WT
WT
MMMa growth, free Psl
ON
E
Lennox growth, bound Psl
ON / OD 0.2 / OD 0.5
WT
WT
Lennox growth, free Psl
ON / OD 0.2 / OD 0.5
MB79
MB79

Figure S4: **A**. Intubation device used for artificial respiration. **B**. Examples of crystal violet stained biofilms formed on a tubing piece of this device, after being incubated with PAO1-3Δ only (left), or successively with PAO1-3Δ (7 hours), then phages (16 hours).

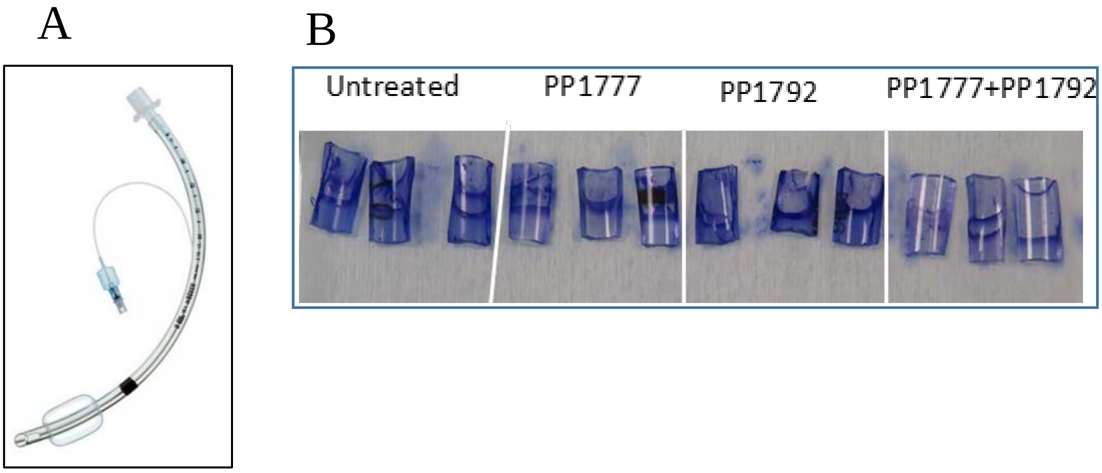

Figure S5: After 6 hours of PAO1_OR biofilm formation at 37°C in a 96-well plate, *LUZ24* phage PP1792 ($5x10^6$ PFU), *LS1* phage PP1450 ($5x10^6$ PFU), or a combination of both (10^7 in total) were added, and the biofilm growth or decay was monitored by confocal microscopy. Each image of the stack was then used to estimate the volume of viable bacteria at each time point, and volumes of living bacteria as a function of time are shown for three technical repetitions on the same plaque.

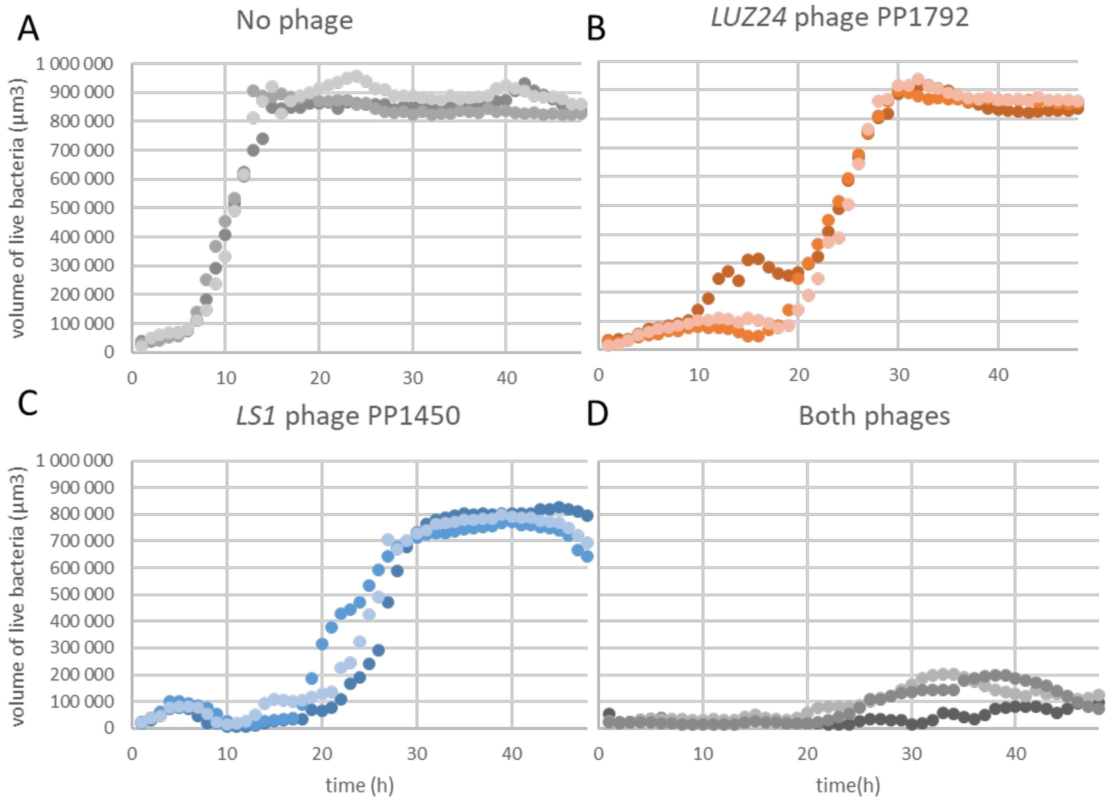

Figure S6: Second biological replicate of Figure 7.

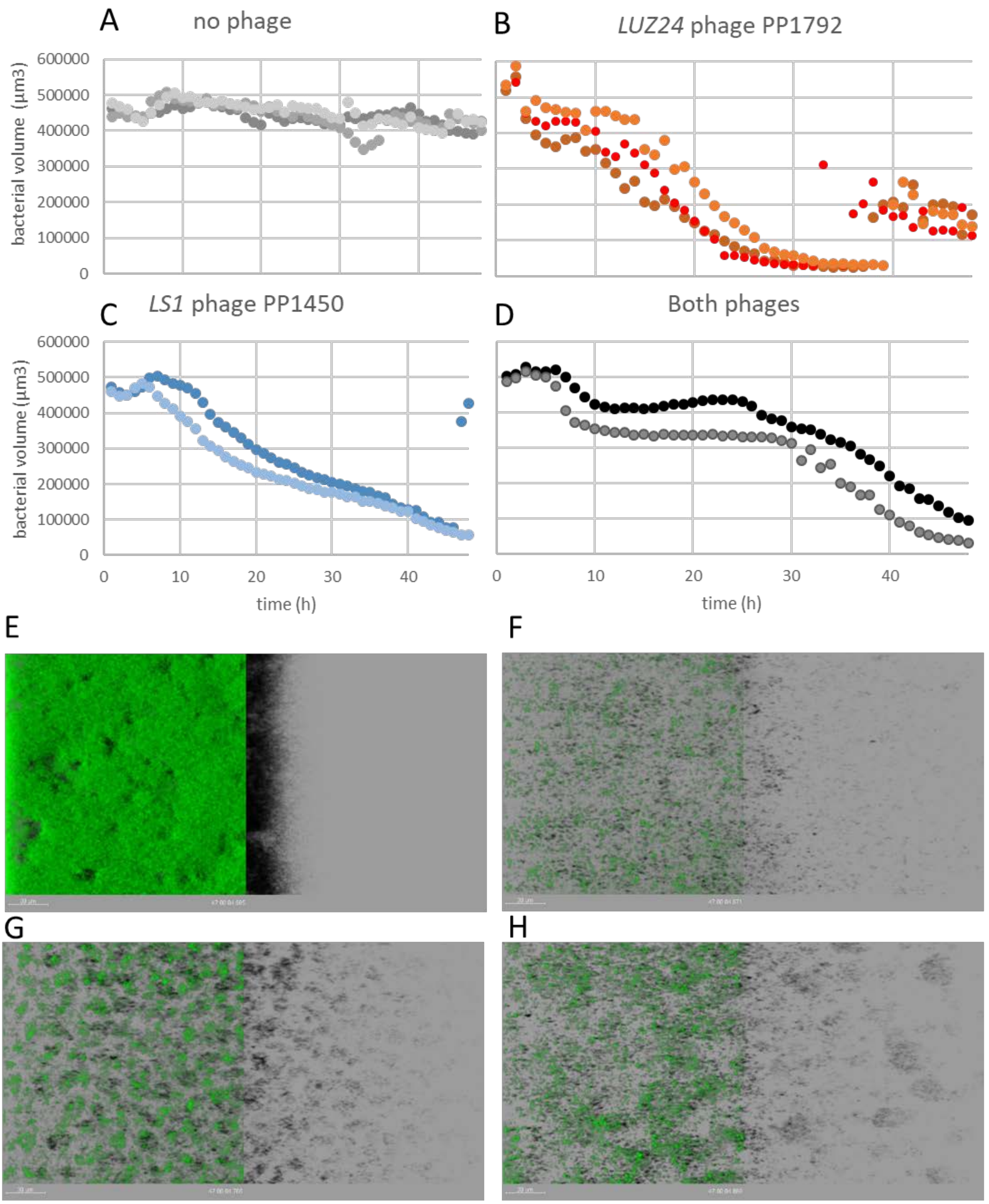

Figure S7: Third biological replicate of Figure 7.

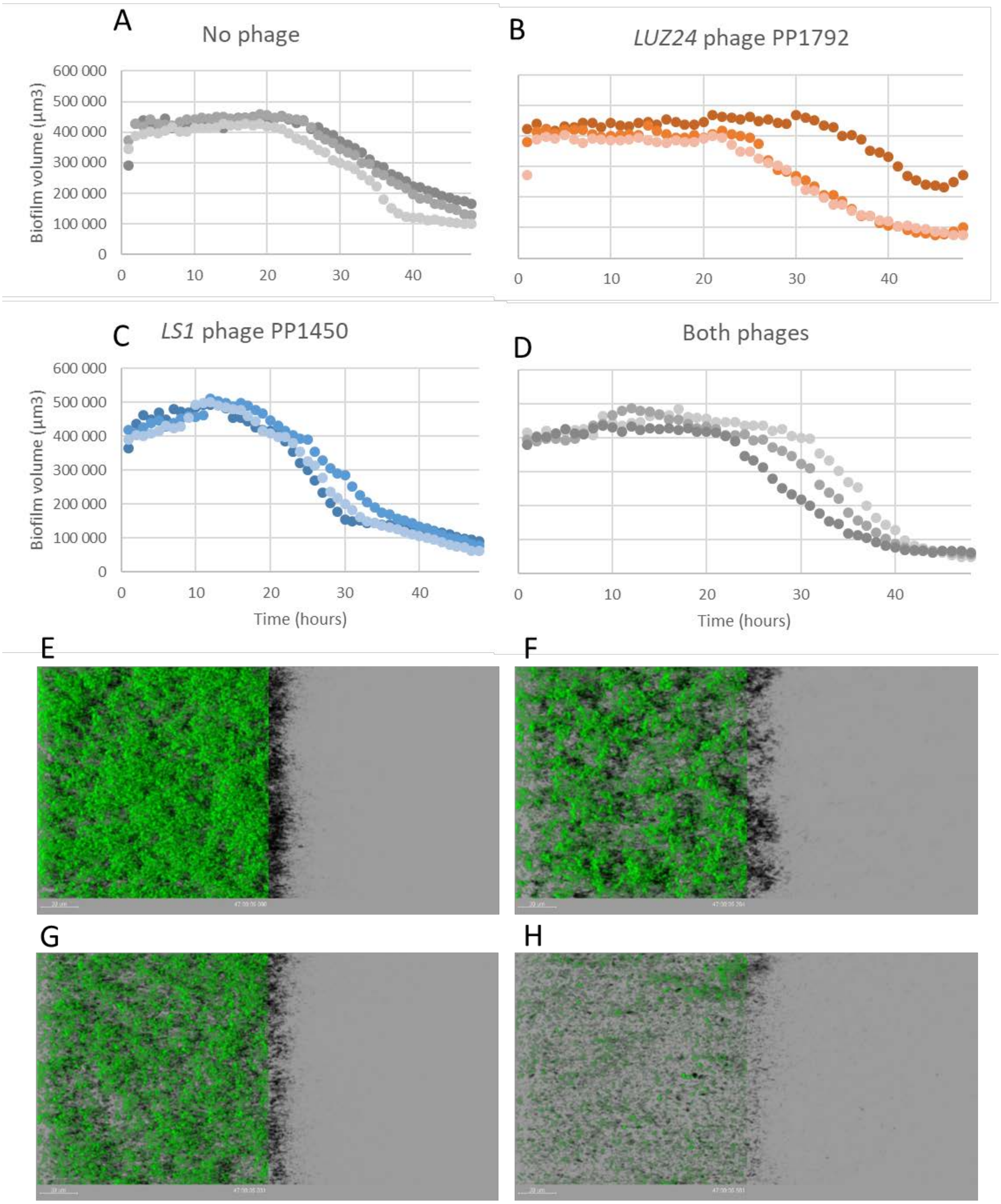

Figure S8: lytic activity of *LUZ24* phage PP1792 on the MB79 *wzy1* mutant strain, in Lennox medium. Growth conditions applied were as described in Figure 2. Phage added at MOI 1 at time 0.

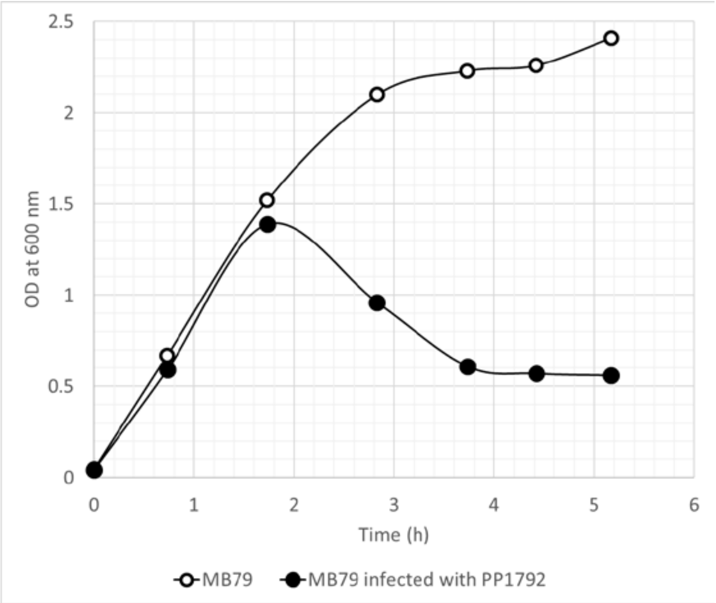

Figure S9: Psl amounts in the MB79 *wzy1* mutant strain, compared to the PAO1-3Δ strain (the PAO1-3Δ values are identical to Figure 5). Bacteria collected from ON cultures, or during exponential growth at OD 0.2 and 0.5 were used to extract surface-bound Psl from equivalent amounts of washed pellets (left side, “bound Psl”), and these extracts were then deposited on a membrane and treated for immunodetection as described in the Methods. In parallel, 5 µL of supernatants from the same cultures (right side, “free Psl”) were directly deposited on the same membranes.

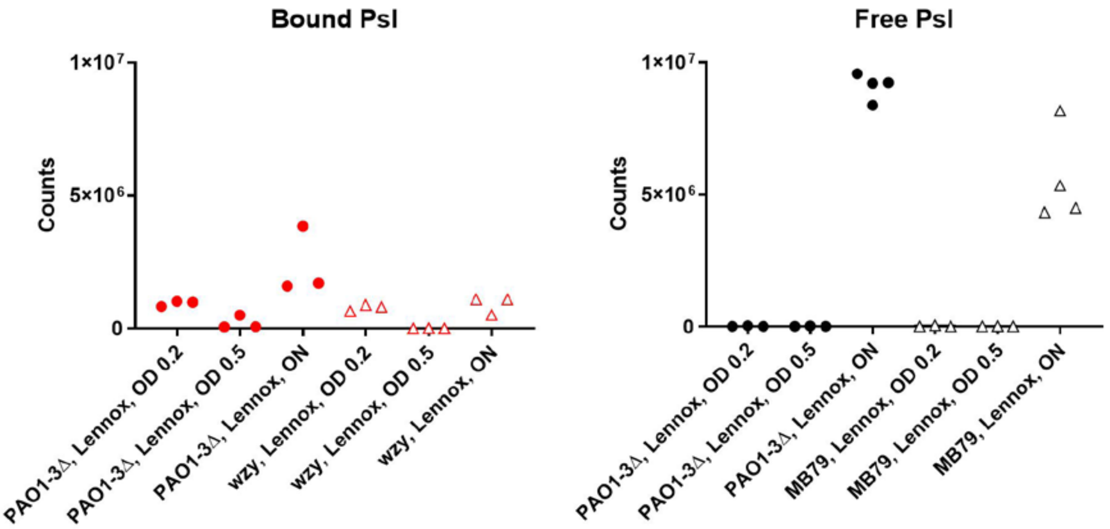

Figure S10. *LUZ24* phage PP1792 adsorption on *wzy1* derivatives in rich medium. Compared to the *wzy1* single mutant, double mutant strains MB87 and MB96 are not affected in their adsorption to phage PP1792 (One way ANOVA, P = 37 and 21%), whereas strain MB99 is an adsorption mutant (P< 0.01%). Adsorption on MB99 is also significantly diminished relative to PAO1-3Δ (P= 0.04%).

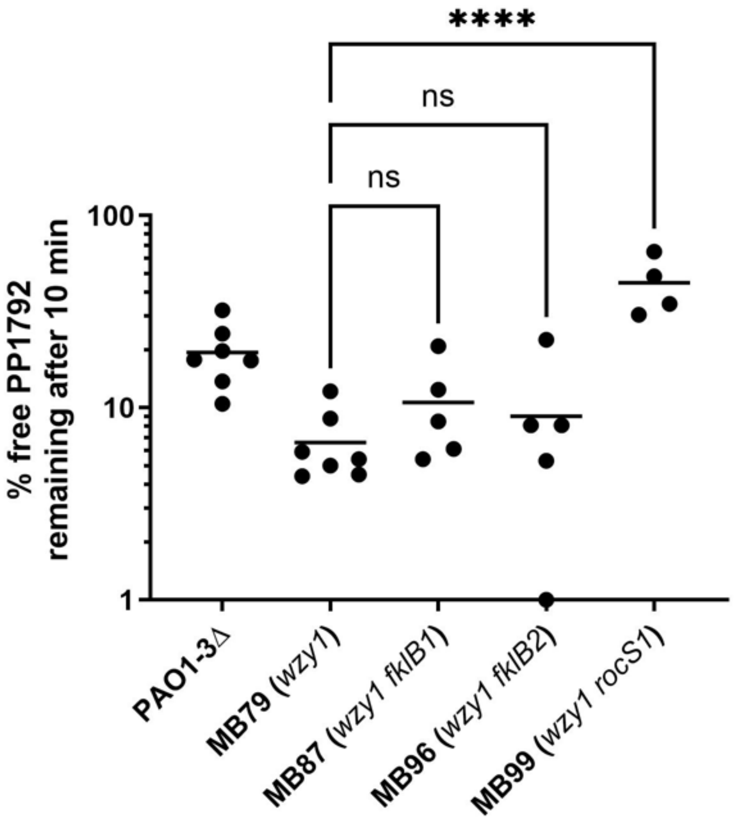

Table S1. Bacteria and plasmids used in this study

Strain or plasmid	Genotype and/ or relevant characteristics	Source or reference
P. aeruginosa PAO1 derivatives		
PAO1_OR		LN871187
PAO1-3Δ	PAO1-OR ΔPf4::FRT Δ*rep*_Pf6::FRT Δ*toxA*	This study
MB75	PAO1-3Δ *wzy2 gloA2*	This study
MB76	PAO1-3Δ *wzy3*	This study
MB078	PAO1-3Δ with 329 240 bp deletion	This study
MB079	PAO1-3Δ *wzy1*	This study
MB132	PAO1-3Δ *pslA1*	This study
MB133	PAO1-3Δ *pslA1*	This study
MB119	PAO1-3Δ *pslD1*	This study
MB153	PAO1-3Δ *pslA2*	This study
MB154	PAO1-3Δ *pslH1*	This study
MB191	PAO1-3Δ pSEVA627M	This study
MB198	PAO1-12228 pSEVA627M	This study
CRR105	PAO1-3Δ Δ(*PAO1OR_2949 –2986*)	This study
CRR107	PAO1-3Δ Δ(*PAO1OR_2949 –2986*)	This study
CRV105	PAO1-3Δ *galU*	This study
CRV109	PAO1-3Δ *PAO1OR_5105*	This study
CMR105	PAO1-3Δ *ygiF1*	This study
MB087	PAO1-3Δ *wzy1 fklB1*	This study
MB096	PAO1-3Δ *wzy1 fklB2*	This study
MB091	PAO1-3Δ *wzy1 fklB1*	This study
MB092	PAO1-3Δ *wzy1 fklB2*	This study
MB099	PAO1-3Δ *wzy1 rocS1*	This study
MB187	PAO1-3Δ *wzy1 wbpL pslH*	This study
MB189	PAO1-3Δ *wzy1 wbpL pslA*	This study
E. coli strains		
β2163	Diaminopurine dependent growth, RP4 conjugation genes inserted into the chromosome	(Demarre et al., 2005)

JM105	*endA*1 *glnV*44 *sbcB*15 *rpsL thi*-1 Δ(*lac-proAB*) [F' *traD36 proAB*+ *lacIq lacZ*ΔM15] *hsdR*4(rK-mK+)	(Yanisch-Perron et al., 1985)
Plasmids		
pPSV35	Shuttle vector with gentamicin resistance gene (*aacCA1*), PA origin, *lacIq*, and *lacUV5* promoter	(Rietsch et al., 2005)
pSEVA627m	Shuttle vector with a msfGFP, RK2 origin, and gentamicin resistance gene	(Silva-Rocha et al., 2013)
pPSVA	*pslA* gene from PAO1-3Δ cloned into pPSV35	This study
pPSVD	*pslD* gene from PAO1-3Δ cloned into pPSV35	This study
pEX18ApGW	Not replicative in *P. aeruginosa*, contains a *sacB* gene and the *oriT* origin.	(Choi and Schweizer, 2005)<br>Accession number AY028469
pEXG2	Not replicative in *P. aeruginosa*, contains a *sacB* gene and an *aac1* gene that is not flanked by FRT sequences.	Accession number KM887143
pFlp2	Contains the flippase gene	Accession number AF048702
pMB01	Integration between *Kpn*I and *Hind*III of pEX18ApGW of a 1660 bp fragment encompassing the PAO1 sequences flanking Pf4, with Pf4 itself being replaced by FRT-*aac1*-FRT. This fragment was amplified on strain PAO1ΔPf4 from (Rice et al., 2009). Left and right flanks are delimited by oligonucleotides (Pf4_BG_5' and 3') and (Pf4BD_3' and 5')	This study
pMB03	Integration between *Kpn*I and *Hind*III of pEX18ApGW of a 1850 bp fragment encompassing the PAO1 sequences flanking the *rep* gene of Pf6, with *rep* itself being replaced by FRT-*aacA1*-FRT. Left and right flanks are delimited by oligonucleotides (Pf6_rep_BG_5' and 3') and (Pf6_rep_BD_3' and 5')	This study
pMB06	Integration between *Eco*RI and *Hind*III of pEXG2 of a 676 bp fragment encompassing the PAO1 sequences flanking the *toxA* gene, placed directly next to each other. Left and right flanks are delimited by oligonucleotides (exoA_BG_5' and 3') and (exoA_BD_3' and 5')	This study

Table S2: Primers used in this study

Name	Description	Sequence
**GmF**	forward primer for the amplification of the gentamicin cassette	CGAATTAGCTTCAAAAGCGCTCTGA
**GmR**	reverse primer for the amplification of the gentamicin cassette	CGAATTGGGGATCTTGAAGTTCCT
**Pf4_BG_5'**	forward primer for the amplification of Pf4 left border (restriction enzyme: KpnI)	CATGGTACCTGGCAGCAGACCCAGGACGC
**Pf4_BG_3'**	reverse primer for the amplification of Pf4 left border	AGGAACTTCAAGATCCCCAATTCGCGTCA TGAGCTTGGGAAGCT
**Pf4_BD_5'**	forward primer for the amplification of Pf4 right border	TCAGAGCGCTTTTGAAGCTAATTCGGATC CCAATGCAAAAGCCCC
**Pf4_BD_3'**	reverse primer for the amplification of Pf4 right border (restriction enzyme: HindIII)	CATAAGCTTTCTGGGAATACGACGGGGGC
**Pf6_rep_BG_5'**	forward primer for the amplification of the left border of Pf6 *rep* gene (restriction enzyme: KpnI)	CATGGTACCAGTAGCGTTGCTCGTGATTG
**Pf6_rep_BG_3'**	reverse primer for the amplification of the left border of Pf6 *rep* gene	AGGAACTTCAAGATCCCCAATTCGGGC GATATGACGGTACGCAAG
**Pf6_rep_BD_5'**	forward primer for the amplification of the right border of Pf6 *rep* gene	TCAGAGCGCTTTTGAAGCTAATTCGATCAT TTGTGCCTCCAG
**Pf6_rep_BD_3'**	reverse primer for the amplification of the right border of Pf6 *rep* gene (restriction enzyme: HindIII)	CATAAGCTTGCTGCGCCTGTTGATCTG
**exoA_BG_5'**	forward primer for the amplification of the left border of *exoA* (restriction enzyme: HindIII)	CATAAGCTTCTCGATAGTCGCCAGGACAC
**exoA_BG_3'**	reverse primer for the amplification of the left border of *exoA* (restriction enzyme: BamHI)	CATGGATCCGAAGGATGAGGCTGATCGAG
**exoA_BD_5'**	forward primer for the amplification of the right border of *exoA* (restriction enzyme: BamHI)	CATGGATCCATCCTGATAGGTAATCCGC
**exoA_BD_3'**	reverse primer for the amplification of the right border of *exoA*	CATGGAATTCGGACATCACCGAGTAGATGC

	(restriction enzyme: EcoRI)	
**pslA_5'**	forward primer for amplification of pslA	TCTGGTACCGCGTTCATCAGTAGACTTCC
**pslA_3'**	reverse primer for the amplification of pslA	TCTGAATTCTCGGCAGAGCAAACAAC
**pslD_5'**	forward primer for amplification of pslD	TCAGGTACCGGCGATGTCGCTCCTCAG
**pslD_3'**	reverse primer for the amplification of pslD	TCAGAATTCCTGGAAGTGAACATCATGAC

Table S3: Efficiency of plating of the four phages on PAO1-3Δ and its *LS1* resistant derivatives, in Lennox. (+) = EOP > 10%; (+/-) = EOP between 10% and 1%; (-/+) = EOP between 1% and 0.01%; (-) = EOP < 10^{-5}.

**Strain**	**Efficiency of plating on Lennox**				**Locus mutated** (PAO1_OR id)	**Nt or amino-acid changes**
	**PP1450**	**PP1777**	**PP1792**	**PP1797**		
PAO1-3Δ	+	+	-	-		
MB79	-	-/+	+	+	*wzy* (1817)	T54*
MB81	-	-/+	+	-/+	*wzy* (1817)	R74*
MB75	-	-/+	-	-	*wzy* (1817), *gloA2* (4299)	Wzy_R74* GloA2_R50H
MB78	-/+	-	-	-	Δ(2768-3055)	329 240 bp deletion (includes *galU*)

Table S4: Area under the curve (AUC) of the Lennox (panel A) and Sputum medium (panel B) growth curves performed in 50 mL volumes (three repetitions), expressed relative to the AUC of the culture that was not phage infected.

A	AUC, relative to uninfected			
Lennox	PP1450	PP1792	Both	Additive *
27/10/2020	0.177	0.796	0.064	0.141
29/10/2020	0.149	0.754	0.052	0.112
23/10/2020	0.178	0.766	0.057	0.136
Average	0.168	0.772	0.057	0.130
Standard deviation	0.017	0.022	0.006	0.015

* expected additive value, if rel AUC (both) ~ rel AUC(PP1450)*rel(AUC PP1792)

B	AUC, relative to uninfected			
Sputum medium	PP1450	PP1792	Both	Additive*
25-nov-20	0.478	0.227	0.115	0.108
06-mai-21	0.773	0.580	0.488	0.448
07-mai-21	0.710	0.416	0.312	0.296
Average	0.654	0.408	0.305	0.284
Standard deviation	0.156	0.176	0.187	0.170

* expected additive value, if rel AUC (both) ~ rel AUC(PP1450)*rel(AUC PP1792)

Table S5: Efficiency of plating of *LUZ24* phages PP1792 and PP1797 on *psl* mutants and complemented strains.

Bacterial strain, mutation	Complementation plasmid	Efficiency of plating	
		PP1792	PP1797
PAO1-3Δ, WT	pPSV35	1	1
MB119, *pslD1*	pPSV35	<10-5	<10-5
MB119, *pslD1*	pPSV-D	1	1
MB132, *pslA1*	pPSV35	<10-5	<10-5
MB132, *pslA1*	pPSV-A	1	1

Table S6: kinetics parameters of biofilm degradation. A time of curve inflexion. B. Slope of degradation after the inflexion time point. The last two columns substract the parameters of biofilms treated with both phages to those of single-phage treatments.

A. inflexion (h)	NO PHAGE	PP1792	PP1450	BOTH	BOTH -1450	BOTH -1792
Figure 7	30	34	20	28		
	29	32	22	20		
	25	28	22	22		
**Average**	**28**	**31**	**21**	**23.3**	10.33	0.33
Figure S6	48	14	10	NO VALUE		
	48	10	7	7		
	48	10	NO VALUE	6		
**Average**	**48**	**11.3**	**8.5**	**6.5**	2	-8
Figure S7	22	30	14	28		
	24	24	16	26		
	22	22	16	23		
**Average**	**23**	**25.33**	**15.33**	**25.67**	-2	-4.83
				**Average**	3.44	-4.17

B. slope (µm3/h)	NO PHAGE	PP1792	PP1450	BOTH	BOTH -1450	BOTH -1792
Figure 7	-5781	-29194	-36250	-29499		
	-8563	-21441	-33931	-21679		
	-9375	-17766	-30243	-41214		
**Average**	-7906	-22800	-33475	-30797	2 677	-7997.00
Figure S6	0	-26218	-10302	NO VALUE		
	0	-17004	-8220	-7251		
	0	-25009	NO VALUE	-8119		
**Average**	0	-22744	-9261	-7685	1 576	15 059
Figure S7	-12184	-16590	-20435	-26852		
	-13600	-18591	-17691	-23457		
	-16770	-16515	-20150	-18357		
**Average**	-14185	-17232	-19425	-22889	-3463.33	-5656.67
				**Average**	263.33	468.33

Table S7. Phage resistant mutants isolated from a co-infection with *LS1* phage PP1777 and *LUZ24* phage PP1792 in Lennox (strains CRR and CRV), or in MMMa (strain CMR).

Bacterial strain	**Efficiency of plating of**				**Locus mutated** (PAO1_OR id)	**Nt or amino acid changes**
	**PP1450**	**PP1777**	**PP1792**	**PP1797**		
CRR105	-	-	-	-	Δ(2949 –2986)	40224 bp deletion (includes *galU*)
CRR107	-	-	-	-	Δ(2949 –2986)	40224 bp deletion (includes *galU*)
CRV105	-	-	-	-	*galU1* (2967)	D130Y
CRV109	-	-	-	-	(5105)	frameshifted protein
CMR105	-	-	-	-	*ygiF1* (5315)	N29H

Table S8: Efficiency of plating of the four phages on mutants resisting to PP1792, starting from strain MB79 (*wzy1*). Mutants were selected either in Lennox (MB87 to MB99) or in MMMa (MB187 and MB189). (+) = EOP > 10%; (+/-) = EOP between 10% and 1%; (-/+) = EOP between 1% and 0.01%; (-) = EOP < 10^{-5}.

Bacterial strain	**Efficiency of plating of**				**Additional locus mutated, relative to MB79** (PAO1_OR id)	**amino acid changes**
	**PP1450**	**PP1777**	**PP1792**	**PP1797**		
MB87	-	+/-	-	-	*fklB1* (4644)	Q74*
MB96	-	+/-	-	-	*fklB2* (4644)	V13D
MB91	-	+/-	-	-	*fklB1* (4644)	Q74*
MB92	-	+/-	-	-	*fklB2* (4644)	V13D
MB99	-	-	+/-	-	*rocS1* (1016)	P724L
MB187	+	+	-	-	*wbpL* (1886)*, *pslH* (2750)	frameshifted PslH
MB189	+	+	-	-	*wbpL* (1886)*, *pslA* (2757)	PslA_R297*

Same mutation in the two clones, WbpL_G92

References

Choi, K.H., and Schweizer, H.P. (2005). An improved method for rapid generation of unmarked Pseudomonas aeruginosa deletion mutants. BMC Microbiol *5*, 30.
Demarre, G., Guerout, A.M., Matsumoto-Mashimo, C., Rowe-Magnus, D.A., Marliere, P., and Mazel, D. (2005). A new family of mobilizable suicide plasmids based on broad host range R388 plasmid (IncW) and RP4 plasmid (IncPalpha) conjugative machineries and their cognate Escherichia coli host strains. Res Microbiol *156*, 245-255.
Rice, S.A., Tan, C.H., Mikkelsen, P.J., Kung, V., Woo, J., Tay, M., Hauser, A., McDougald, D., Webb, J.S., and Kjelleberg, S. (2009). The biofilm life cycle and virulence of Pseudomonas aeruginosa are dependent on a filamentous prophage. ISME J *3*, 271-282.
Rietsch, A., Vallet-Gely, I., Dove, S.L., and Mekalanos, J.J. (2005). ExsE, a secreted regulator of type III secretion genes in Pseudomonas aeruginosa. Proc Natl Acad Sci U S A *102*, 8006-8011.
Silva-Rocha, R., Martinez-Garcia, E., Calles, B., Chavarria, M., Arce-Rodriguez, A., de Las Heras, A., Paez-Espino, A.D., Durante-Rodriguez, G., Kim, J., Nikel, P.I., *et al.* (2013). The Standard European Vector Architecture (SEVA): a coherent platform for the analysis and deployment of complex prokaryotic phenotypes. Nucleic Acids Res *41*, D666-675.
Yanisch-Perron, C., Vieira, J., and Messing, J. (1985). Improved M13 phage cloning vectors and host strains: nucleotide sequences of the M13mp18 and pUC19 vectors. Gene *33*, 103-119.
